# Metabolic Profiling of Human Plasma and Urine, Targeting Tryptophan, Tyrosine and Branched Chain Amino Acid Pathways

**DOI:** 10.3390/metabo9110261

**Published:** 2019-11-01

**Authors:** Andrea Anesi, Josep Rubert, Kolade Oluwagbemigun, Ximena Orozco-Ruiz, Ute Nöthlings, Monique M.B. Breteler, Fulvio Mattivi

**Affiliations:** 1 Department of Food Quality and Nutrition, Research and Innovation Centre, Fondazione Edmund Mach (FEM), Via E. Mach 1, 38010 San Michele all’ Adige, Italy; 2 CIBIO, Department of Cellular, Computational and Integrative Biology, Via Sommarive 9, 38123 Povo, Italy; 3 Nutritional Epidemiology, Institute of Nutrition and Food Sciences, University of Bonn, Endenicher Allee 19b, 53115 Bonn, Germany; 4 Population Health Sciences, German Center for Neurodegenerative diseases (DZNE), Venusberg-Campus 1-Building 99, 53127 Bonn, Germany; 5 Institute for Medical Biometry, Informatics and Epidemiology (IMBIE), Faculty of Medicine, University of Bonn, Venusberg-Campus 1-Building 11, 53127 Bonn, Germany; 6 University of Trento, Department of Physics, Bioorganic Chemistry Laboratory, Via Sommarive 14, 38123 Povo, Italy

**Keywords:** tryptophan metabolism, tyrosine metabolism, branched chain amino acids, gut microbiota metabolites, targeted metabolomics, LC-MS/MS, human plasma, urine, clinical studies

## Abstract

Tryptophan and tyrosine metabolism has a major effect on human health, and disorders have been associated with the development of several pathologies. Recently, gut microbial metabolism was found to be important for maintaining correct physiology. Here, we describe the development and validation of a UHPLC-ESI-MS/MS method for targeted quantification of 39 metabolites related to tryptophan and tyrosine metabolism, branched chain amino acids and gut-derived metabolites in human plasma and urine. Extraction from plasma was optimised using 96-well plates, shown to be effective in removing phospholipids. Urine was filtered and diluted ten-fold. Metabolites were separated with reverse phase chromatography and detected using triple quadrupole MS. Linear ranges (from ppb to ppm) and correlation coefficients (*r*^2^ > 0.990) were established for both matrices independently and the method was shown to be linear for all tested metabolites. At medium spiked concentration, recovery was over 80% in both matrices, while analytical precision was excellent (CV < 15%). Matrix effects were minimal and retention time stability was excellent. The applicability of the methods was tested on biological samples, and metabolite concentrations were found to be in agreement with available data. The method allows the analysis of up to 96 samples per day and was demonstrated to be stable for up to three weeks from acquisition.

## 1. Introduction

The emerging field of “nutrition-microbiome-human health” has raised many unanswered questions regarding the complex relationship and interplay of gut microbiota, their metabolites in homeostasis and human physiology. Results from epidemiological studies, clinical trials and recent meta-analyses have supported the link between mood disorders, obesity and gut microbiota [1,2,3], and recent data have strikingly indicated that emotional stress, anxiety and depression may influence the development of gastrointestinal disorders and cancer [4,5,6,7,8]; however, the relationship between them is still unclear. It has been suggested that gut microbial metabolites act on the gut epithelium, modulating downstream signalling pathways involved in the control of digestion, metabolism, immunity, the brain and pain [9,10,11,12,13].

Indeed, during the last few years, the link between gut microbiota and the brain has been investigated in depth [3,14,15,16], demonstrating that depression is associated with decreased gut microbiota richness and diversity [17]. The concept of the “brain-gut-microbiome axis” has recently been introduced to describe the complex interactions between gut microbiota and human physiology [18].

**Figure 1:**
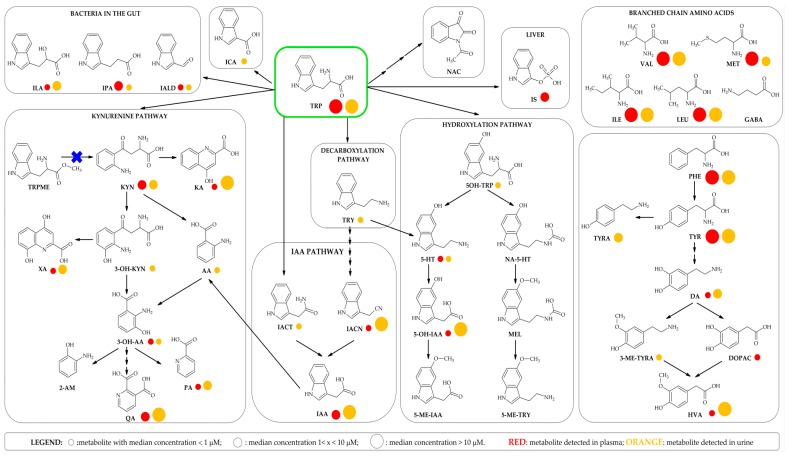
Principal branches of the TRP and TYR metabolic pathways covered in this analytical method and structures of the main BCAAs. Red circles represent the metabolites detected in plasma, orange circles those detected in urine. The size of the circle is proportional to the median concentration in each biofluid. 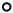: metabolite with median concentration of < 1 μM; 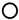: metabolite with median concentration of 1 < x < 10 μM; 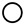: metabolite with median concentration of > 10 μM.

Tryptophan (TRP) metabolism has claimed to be a key player in neurophysiology and depression [14,17,18,19,20], regulation of immune response and inflammation, inflammatory bowel diseases [21], metabolic syndrome and obesity [18]. TRP is an essential amino acid bearing an indole group critical for protein synthesis, but it also serves as a substrate for the generation of several bioactive compounds. In mammals, about 95% of ingested TRP is catabolised through the kynurenine pathway (KP) [20,22,23] into a range of metabolites known to be involved in inflammation, immune response and excitatory neurotransmissions [22,24] (Figure 1). Kynurenine (KYN) and its metabolites are known for their beneficial effects on the central nervous system [20].

Minor pathways of TRP degradation lead to synthesis of the neurotransmitter serotonin (5-HT) via the hydroxylation pathway, tryptamine (TRY) via decarboxylation and indole-3-pyruvic acid via transamination pathways [11,23]. Several bacteria have the ability to synthesize 5-HT from dietary TRP, and are therefore able to modulate the brain-gut axis [25]. Gut microbiota can also produce indole and its derivatives, such as indole-3-propionic acid (IPA), indole-3-lactic acid (ILA) and indole-3-aldehyde (IALD) [26]. Indole is synthesized from TRP via the tryptophanase enzyme and this metabolite is able to maintain host-microbe homeostasis on the mucosal surface [26,27,28]. Hepatic sulfonation of indole leads to the production of indoxyl sulfate (IS), a cytotoxic metabolite that induces renal and vascular dysfunction [29,30]. IPA is a potent antioxidant able to reduce DNA damage and lipid peroxidation, and to maintain mucosal homeostasis and intestinal barrier functions [31,32]. *Clostridium sporogenes* is the predominant IPA producer, but a recent work demonstrated that four other gut bacteria can synthesize it: *Peptostreptococcus anaerobius* CC14N and three strains of *Clostridium cadaveris* [33]. ILA is an intermediate of IPA production from TRP operated by *C. sporogenes,* but it is also produced by *Bifidobacterium* spp [34]. ILA was also identified as a potential biomarker for alcohol-induced liver disease in Ppara mouse model [35]. IALD is produced from bacteria belonging to the *Lactobacillus* genera and helps to maintain host-microbial homeostasis [36].

The importance of qualitatively and quantitatively understanding gut microbiota regulation of TRP metabolism in healthy and diseased conditions thus appears to be clear. On the other hand, emerging evidence has also shown that different concentrations in human biofluids (blood and urine) and tissues of branched chain amino acids (BCAAs), such as L-methionine (MET), L-valine (VAL), L-isoleucine (ILE) and L-leucine (LEU) among others, might play an unrecognised and crucial role in the development of intestinal health [37,38], depression [39] and cancer [40]. In the light of these facts, it is clear that there is a complex inter-kingdom regulatory network and interactions occurring between the host, microbiome, and diet.

Accurate quantitation of TRP derived metabolites and BCAAs in plasma, serum and urine is becoming increasingly important, since subtle changes may be responsible for mechanistic responses. However, to date the development and validation of a single robust targeted method providing broad coverage and suitable for the main biofluids is still lacking. More frequently, only a few metabolites related to KP have been monitored [41,42,43,44,45,46,47,48]. Zhu and colleagues reported on the quantitation of 19 metabolites in urine and human serum, including microbial derived metabolites [49]. One main drawback of this method, which could limit applicability in clinical studies requiring the inclusion of a large number of samples, is the number of laborious steps proposed for metabolite extraction, which requires the use of single tubes, 1 h incubation at −20 °C to ensure protein precipitation and three centrifugation steps.

In 2016, Marcos and co-workers proposed a method for quantitation of 17 TRP metabolites and BCAAs in urine and plasma [50]. Again, the procedure for extraction of metabolites from plasma limited the processing of a large sample set. More recently, Whiley and colleagues (2019) published a thoroughly validated method for the quantitation of 18 TRP metabolites in serum and plasma based on Phenomenex PHREE SPE 96-well plate extraction that allows high-throughput sample preparation [51]. The study, which to our knowledge represents the state-of-the-art in the field, covers the quantitative analysis of 18 metabolites associated with KP and 5-HT degradation pathways, but did not cover BCAAs or gut-derived metabolites.

Here, we present a validated analytical method for the simultaneous separation and detection of 39 metabolites in both plasma and urine using Ultra High Performance Liquid Chromatography-ElectroSpray-Ionization-Tandem Mass Spectrometry (UHPLC-ESI-MS/MS). Preliminary application of this method in two independent epidemiological studies across the lifespan of the DOrtmund Nutritional and Anthropometric Longitudinally Designed (DONALD) Study and the Rhineland Study allowed us to establish the typical ranges for these 39 metabolites present in the human biofluids of two German populations.

Legend: TRP: L-tryptophan; KYN: kynurenine; KA: kynurenic acid; 3-OH-KYN: 3-hydroxy kynurenine; 3-OH-AA: 3-hydroxy-anthranilic acid; AA: anthranilic acid; XA: xanthurenic acid; QA: quinolinic acid; PA: picolinic acid; 2-AM: 2-aminophenol; 5-OH-TRP: 5-hydroxy-L-tryptophan; 5-HT: serotonin; 5-OH-IAA: 5-hydroxyindole-3-acetic acid; 5-ME-IAA: 5-methoxyindole-3-acetic acid; NA-5-HT: *N*-acetyl-5-hydroxytryptamine; MEL: melatonin; 5-ME-TRY: 5-methoxytryptamine; TRY: tryptamine; IACN: indole-3-acetonitrile; IAA: indole-3-acetic acid; IACT: indole-3-acetamide; ILA: indole-3-lactic acid; IPA: indole-3-propionic acid; IALD: indole-3-carboxaldehyde; IS: indoxyl sulfate; NAC: 1-acetylisatin; ICA: indole-3-carboxylic acid; TRPME: tryptophan methyl ester; PHE: phenylalanine; TYR: tyrosine; TYRA: tyramine; DA: dopamine; 3-ME-TYRA: 3-methoxy-p-tyramine; DOPAC: 3,4-dihydroxyphenyl acetic acid; HVA: homovanillic acid; GABA: gamma-aminobutyric acid; ILE: L-isoleucine; LEU: L-leucine; VAL: L-valine; MET: L-methionine.

## 2. Results

### 2.1. Liquid Chromatography and Mass Spectrometry

Two Multiple Reaction Monitoring (MRM) transitions were optimised for each target compound by changing Collision Energy (CE) and Cone Voltage (CV). The one displaying the highest intensity was selected as the quantifier ion (Q), while the less intense one was selected as the qualifier ion (q). MS parameters and retention times (RT) are reported in Table 1.

**Figure 2:**
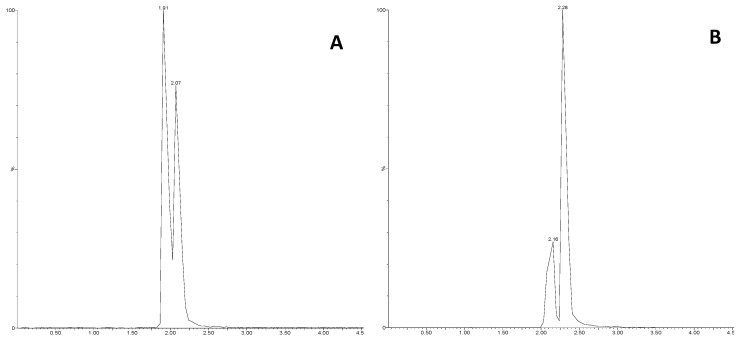
MRM (132.096 > 86.0) for ILE (left peak) and LEU (right peak) in plasma samples. (**A**): Waters ACQUITY BEH C_18_ 1.7 μm, 2.1 × 150 mm; (**B**): Waters ACQUITY HSST3 1.8 μm, 2.1 × 150 mm.

With our chromatographic setup, GABA was practically non-retained and eluted with the chromatographic front. The 150 mm column enabled separation of ILE from LEU, as highlighted in Figure 2 for the BEH (panel A) and HSST3 (B) columns. The HSST3 column provided baseline separation of ILE (RT: 2.25 min) from LEU (RT: 2.38 min).

The presence of different substituents on the indole moiety enabled separation of all indole derivatives within 8.5 min. The total run time, including column re-equilibration, was 14 min. This made it possible to acquire up to 96 samples (one 96-well plate) in 24 h. Directing flow waste during non-acquisition time enabled us to acquire up to 300 samples per batch without significant signal losses for both matrices.

### 2.2. Linearity and Limit of Quantification (LOQ)

The linearity range for each metabolite was established by using calibration curves in water with 0.1% formic acid (FA), since it was impossible to obtain analyte-free matrices (blanks). Linearity ranges covered 4+ orders of magnitude, from a few ppb to ppm. The availability of a large number of samples obtained from two independent German observational and epidemiological studies across the lifespan allowed us to finely tune calibration. Working calibration ranges were specifically designed for each metabolite, to cover the expected concentrations in plasma and urine. All working calibration curves were found to have a good correlation coefficient (*r*^2^ > 0.990) in the tested ranges for both plasma and urine (see Table S1).

Metabolites at low concentration levels were linear in the range 1–250 ng/mL. By contrast, for high level metabolites, the upper quantification point was 12500 ng/mL, and above this the MS response was no longer linear. The exceptions were 3-hydroxykynurenine (3-OH-KYN) in urine, which was linear between 15–25,600 ng/mL, and homovanillic acid (HVA) in plasma, which was linear in the range of 156–25,000 ng/mL.

LOQs were in the order of a few ng/mL for low level metabolites, except for 2-aminophenol (2-AM) (78.1 ng/mL) and 3-hydroxyanthranilic acid (3-OH-AA) (31.2 ng/mL) in plasma, indole-3-acetonitrile (IACN) in urine (19.5 mg/mL) and 3-OH-KYN in both matrices (31.2 and 15.6 ng/mL in plasma and urine respectively). For high level metabolites, the LOQ was set as the lowest calibration point. Metabolites detected in negative ion mode (HVA, DOPAC and IS) displayed higher LOQs in both matrices.

### 2.3. Retention Time Stability

For both matrices, metabolite RT stability was addressed over a period of three weeks. Most of the metabolites showed a coefficient of variation lower than 1%, except for 2-AM (CV%: 2.10) and TYR-d4 (CV% 1.04) in plasma, and IS (CV% 1.09) in urine. See Table S1 for details on plasma and urine respectively.

### 2.4. Matrix Effects

Matrix effects (ME), evaluated with the matrix match calibration (MMC) approach, were minimal and in the range of 80–120% for most metabolites in both plasma and urine (Table S1). Ion suppression by the matrix component significantly affected quantification of the most polar metabolites: VAL, dopamine (DA), MET, and quinolinic acid (QA) were suppressed both in plasma and urine, while 2-AM and tyramine (TYRA) were affected only in urine. Quantification of GABA was significantly deviated in both matrices due to its poor retention with a C_18_ analytical column.

### 2.5. Recovery, Intra- and Inter-Day Accuracy and Precision

At medium spiked concentration, metabolite recovery from plasma was over 85%, except for VAL (80.7%) and picolinic acid (PA) (82.8%). At low spiked concentration, recovery was over 80% for all metabolites except GABA (71.2%) and QA (76.5%). In urine, recovery at medium spiked concentration was over 80% for all metabolites. At low spiked concentration, several metabolites, such as DA, ILE, LEU, TYRA and 3-OH-KYN, had lower recovery, due to the fact that spiked values were close to the LOQ, so analytical error was greater. Recovery at the highest spiked concentration was slightly over 80% for all metabolites, except 3-OH-AA in urine and GABA in both matrices. All information on plasma and urine can be found in Table S2.

Accuracy at medium spiked concentration was excellent for all metabolites (CV<15%) in both plasma and urine (Table S2). At low concentrations, precision was lower than 20% for all metabolites in plasma, except for PA, 3-OH-KYN, 3-methoxy-p-tyramine (3-ME-TYRA), 3-OH-AA, DOPAC, 5-methoxytryptamine (5-ME-TRY), 5-hydroxyindole-acetic acid (5-OH-IAA) and TRP- methyl ester (TRPME). Accuracy at low spiked concentration was not calculated for the internal standard. At the highest concentration, accuracy was low for VAL in plasma and PHE, TYR, 3-OH-AA and TRP in urine. This was due to the fact that spiked amounts were above the detector linear response. Accuracy was unsatisfactory for GABA at all concentration levels and was not reported. We propose to use data on GABA to detect fold changes rather than to provide accurate quantitative data.

### 2.6. Carryover Effect and Phospholipid Removal

**Figure 3:**
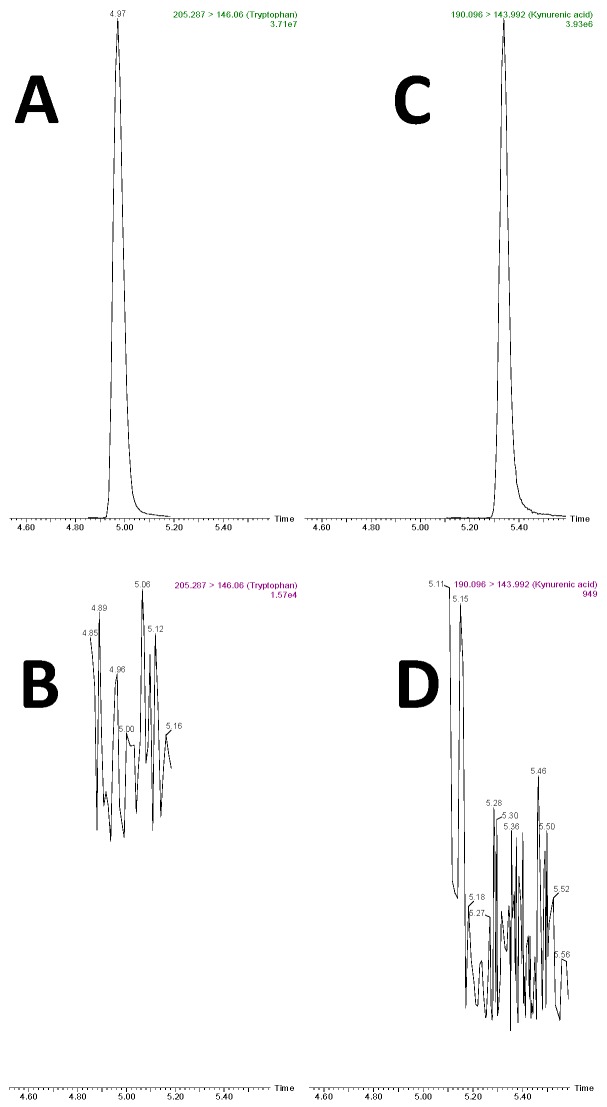
MRM of TRP (205.29 > 146.06) after injection of a plasma sample spiked at the highest concentration (panel **A**) and injection of ACN at the end of the entire batch (*n* = 5) (**B**). MRM of KA (190.09 > 143.99) at the highest point of calibration (**C**) and in the following run after injection of ACN (**D**).

No carryover effect was observed within and between runs for either plasma or urine. Figure 3 shows the MRM transition of TRP (205.29 > 146.06; RT: 4.95 min) for a plasma sample spiked at the highest concentration (25,000 ng/mL) (panel A) and the blank, after the acquisition of 5 plasma samples spiked at the highest concentration (B). Similarly, (C) shows the MRM transition of kynurenic acid (KA) (190.09 > 149.99; RT: 5.45 min) at the highest calibration point, while (D) shows the MRM acquired in the following run after injection of acetonitrile (ACN). Column cleaning for 3 min at 100% B ensured complete elution of the tested metabolites, while the strong wash solvent ensured good needle cleaning.

**Figure 4:**
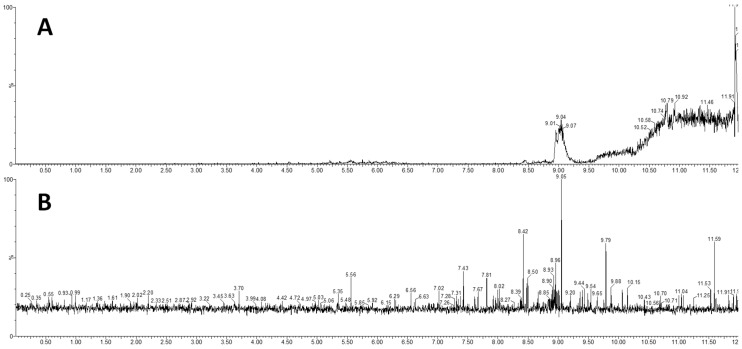
Panel **A**: Chromatogram of PIS of *m/z* 184.03 in untreated plasma, showing the signal generated by presence of PC and SM. Panel **B**: chromatogram of PIS of *m/z* 184.03 after plasma clean up on an Ostro 96-well plate, demonstrating the removal of interfering signal due to lipids eluting after 8.50 min

Biological samples, particularly plasma, contain significant amounts of phospholipids, mainly phosphatidylcholine (PC), phosphatidylethanolamine (PE) and sphingomyelin (SM). All these matrix components can significantly affect compound ionization through ion enhancement/suppression effects. With our chromatographic setup, phospholipids eluted after 8.50 min, therefore well after the last eluting metabolite (IPA, RT: 8.06 min). Nevertheless, Ostro 96-well plates were also able to efficiently remove phospholipids from plasma, as demonstrated by the Precursor Ion Scan (PIS) of *m/z* 184.03 on crude plasma (Figure 4A) or plasma after sample clean up (B). Urine contained traces of PC and SM and 10-fold dilution did not affect the MS response (data not shown).

### 2.7. Method Application to Biological Samples

To demonstrate the applicability of the method, we analysed fasting samples of plasma (*n* = 1000) and 24-h urine samples (*n* = 672) from two independent populations. As the Rhineland Study did not collect 24-h urine and the DONALD study did not collect blood, no paired samples were available from the same individual. Samples were pseudonymised and randomised prior to extraction, and were extracted independently. Biological QCs were prepared by mixing equal volumes of sample. Twenty QCs were injected at the beginning of the acquisition sequence in order to stabilise the MS response and at intervals of 15 samples across the sequence in order to test MS stability. Calibration curves were acquired after the first 20 QCs, approximately every 300 samples, and at the end of each batch.

**Figure 5:**
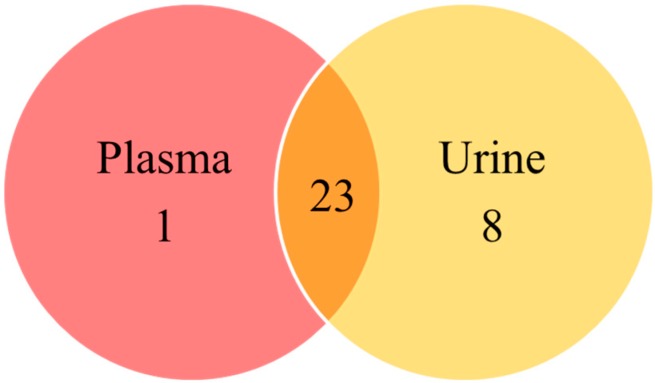
Venn diagram illustrating metabolites found exclusively in plasma (1), exclusively in urine (8) and in both matrices (23).

The method allowed quantification of 24 metabolites in plasma and 30 metabolites in urine: 23 metabolites were common to the two matrices; DOPAC was detected exclusively in plasma, while 8 metabolites were found exclusively in urine (Figure 5). The metabolites in our study resulting unique to urine were the neurotransmitter TYRA, the metabolite from methylation of DA, 3-ME-TYRA, the immediate precursor of 5-HT, 5-OH-TRP, the highly reactive neurotoxin 3-OH-KYN and the uremic toxin AA in the KP, as well as the β-arylamine neurotransmitter and microbial catabolite TRY and the intermediate of the indole-3-acetic acid (IAA) pathway indole-3-acetamide (IACT) (Figure 1).

**Figure 6:**
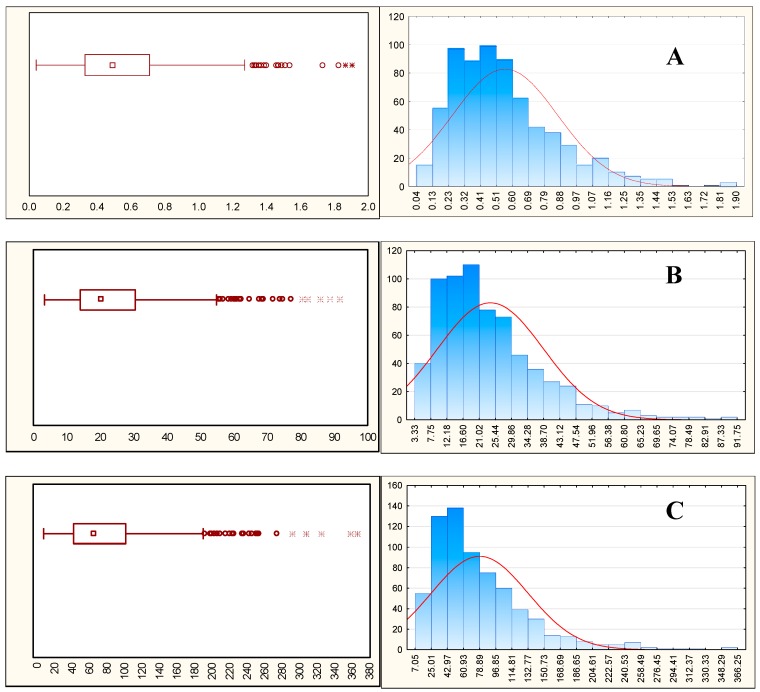
Box plots (90% confidence interval) and graphic distribution of metabolites with (**A**) low (5-HT, median 0.492 μM), (**B**) medium (XA, median 5.424 μM) and (**C**) high median (TRP, median 64.02 μM) levels quantified in urine in the DONALD study (*n* = 672).

The minimum, median and maximum values detected in both matrices for the two German populations are reported in Table 2. Qualitatively, here we divided the metabolites into three categories according to the median value: low (median < 1 µM), medium (median 1< × <10) and high (median > 10) level metabolites (Figure 6). In general, high level metabolites in plasma (VAL, ILE, LEU, MET, TYR, PHE and TRP) were also present in high concentrations in urine. Medium level metabolites (QA, KYN, IACN and IAA) were detected in higher concentrations in urine, except for IPA, which was found at a lower concentration. Of the low level metabolites in plasma, XA, KA, 5-OH-IAA and HVA were those excreted at the highest concentrations in urine. As expected, indole-3-carbocylic acid (ICA) was detected only in urine.

## 3. Discussion

### 3.1. Optimisation of MS Parameters and Analytical Specificity

The selection of Q and q ions was based on signal intensity, with Q ions being the most intense. In order to increase MS settling time, we selected just one qualifier ion per molecule and tried to avoid MRM transitions common to many metabolites when possible. Recently, Whiley et al. (2019) highlighted that the second transition of the TRP 13C-isotope shares the same MRM transition as XA (206.09 > 132.01), interfering with its quantification [51]. With our experimental setup, XA quantification was achieved by MRM transition (206.09 > 160.01) while the MRM (206.09 > 132.01) was used for qualitative purposes. Furthermore, the two peaks were chromatographically sufficiently separated, having TRP and XA RT of 4.94 and 5.03 min respectively. The MRM transition (206.09 > 160.01) was also common to ILA (RT: 6.96 min) and 5-methoxyindole-acetic acid (5-ME-IAA) (RT: 7.35 min), but the three molecules were well separated.

Similarly, *N*-acetyl-5-hydroxytryptamine (NA-5-HT) and the TRPME had the same MRM for quantification (219.2 > 160.0), but the two peaks were baseline separated, having an RT of 5.86 and 6.07 min respectively.

All the tested compounds except DOPAC and HVA contained nitrogen atoms and were easily detected as [M+H]^+^ in positive ion mode. IS contains both a nitrogen atom and a sulfate group, but a better response is obtained in negative ion mode. Therefore DOPAC, HVA and IS were detected as [M–H]^-^, by setting up polar switching within the chromatographic run.

Calibration accuracy was obtained with the use of 8 deuterated standards (Table 1). Since deuterated internal standards were not available for all the tested metabolites, and in order to limit the cost of the calibration, we opted for a) chemically related molecules (i.e., TYR-d4 for PHE, TRP-d5 for indole derivatives and DOPAC-d5 for HVA in negative ion mode) and b) molecules eluting nearby (i.e., MET-d4 for BCAAs).

### 3.2. Choice of Chromatographic Technique

Two analytical approaches were initially tested, based on Reversed-Phase (RP) chromatography on a Waters ACQUITY BEH C_18_ 1.7 μm, 2.1 × 150 mm, and Hydrophilic Interaction Liquid Chromatography (HILIC) using a Waters ACQUITY BEH AMIDE 1.7 μm, 2.1 × 150 mm. HILIC was tested due to the high polarity of certain metabolites, such as GABA, VAL, ILE, LEU, TYR, MET, DA, among others. This column is widely considered to be suitable for the analysis of several other polar metabolites [52]. This column provided excellent efficiency and chromatographic resolution for separation of most polar compounds, but indole derivatives were poorly retained, hampering their separation and quantification (see Figure S1 for details).

HILIC RT stability was affected by slight pH modification and we noticed that RT shifts could appear during long acquisition sequences. Nevertheless, ammonium formate, present in both mobile phases, tends to stick on the orifice plate after desolvation, which increases the chance of source contamination and ion suppression. All these considerations made HILIC relatively less attractive for the analysis of TRP-derived metabolites in a large number of biological samples. At this point, RP chromatography was selected for further method optimisation and HILIC remained as a complementary tool for the separation of highly hydrophilic compounds.

### 3.3. Optimisation of Chromatography on C_18_ Stationary Phase

Five different RP columns were tested using different elution programs: Waters ACQUITY BEH C_18_ 1.7 μm, 2.1 × 150 mm; Waters ACQUITY HSST3 1.8 μm, 2.1 × 150 mm; Waters Cortecs UPLC C_18_ 1.6 μm, 2.1 × 100 mm; Phenomenex Kinetex Polar C_18_ 2.6 μm, 2.1 × 100 mm; and Phenomenex Kinetex EVO C_18_ 2.6 µm, 2.1 × 100 mm (see Figure S2 for details).

Most polar compounds were poorly retained on 100 mm columns, even if the percentage of aqueous solvent was increased to 100%; therefore, these columns were not selected for further optimisation. 150 mm columns demonstrated the same separation efficiency, but the Waters HSST3 column was selected given that a) polar compounds are better retained, b) the critical couple of analytes ILE (RT: 2.25 min) and LEU (RT: 2.38 min) are baseline separated (Figure 2, panels A and B).

### 3.4. Comparison of Proposed Extraction Procedures and Analytical Performance: Efficiency and Efficacy

The efficiency and efficacy of Ostro 96-well plate and Liquid-Liquid Extraction (LLE) methods were first evaluated for an initial set of 21 key metabolites in plasma, by studying recovery and relative standard deviations (RSDs) (Table 3). The Ostro 96-well plate rapidly extracted and precipitated proteins using ice-cold ACN, containing 1% FA. To improve metabolite recovery, plates were shaken twice for 10 min before filtering; different ratios of water: ACN (1:1, *v*/*v*, 8:2, *v*/*v* and pure ACN), and the addition of FA were also tested as reconstitution solvents. According to the manufacturer’s procedures, a mixture of water and MeOH were used to recover extracted metabolites. In our case, MeOH was replaced by ACN in order to a) increase retention and b) increase the selectivity and peak shapes of more polar compounds, especially those eluting in the first 3 min of the chromatographic run. Ultimately, water: ACN (8:2, *v*/*v*) 0.1% FA was a good compromise for metabolite recovery, peak shape and chromatographic selectivity. On the other hand, urine samples were simply diluted and filtered. In this context, the dilution factor was first studied. Urine samples were diluted five- and ten-fold in water with 0.1% FA. Five-fold dilution enabled the detection of metabolites present in low amounts as DA, but those present at high levels could saturate the detector, hampering quantification. Ten-fold dilution avoided detector saturation, while at the same time the presence of more water in the sample improved the retention of polar compounds and peak shapes.

LLE and Ostro 96-well plate methods were compared in terms of plasma recovery (Table 3). In this table, LLE and Ostro 96-well plate showed a suitable range of recovery, which in the vast majority of cases was over 60%. However, the LLE method showed relatively lower recovery rates compared to Ostro 96 well plate, ranging from 50% to 90%. Recovery was over 75% for LEU, ILE and VAL. By contrast, 5-OH-TRP and KA did not reach 65% recovery, and XA showed the lowest recovery. On the other hand, Ostro 96-well plate was able to adequately extract the selected metabolites, with 15 metabolites ranging from 90-100%, such as TRP, MET and KYN. More importantly, the recovery obtained was in an acceptable range and RSDs were below 20%. To sum up, data comparison showed that the Ostro 96-well plate method offered an appropriate range of recovery and low RSDs compared with LLE. It should be noted that this method is supposed to be routinely used within the HEALTHMARK project and thousands of samples from several clinical studies would be analysed. Therefore, in addition to the standard parameters required for a new method (sufficiently innovative and robust compared to other available methods for the intended application), here reproducibility, speed and accurate quantification are strongly required. For these reasons, the Ostro 96-well plate method was selected, since it was the most efficient and effective extraction procedure evaluated. The combination of sample preparation with an Ostro 96-well plate and UHPLC separation with C_18_ stationary phase was selected for further studies in order to extend the number of metabolites and validate the method.

### 3.5. Method Validation

The following parameters were studied for 40 metabolites related to TRP and TYR metabolism and BCAAs: linearity, LOQ, recovery, precision as repeatability and within-lab reproducibility, process efficiency and ME. Calibration curves were designed independently for plasma and urine to cover the expected metabolite concentration range according to available data, and changed accordingly after the analysis of biological samples, in order to precisely define the typical working range for each metabolite.

Linear dynamic ranges for both plasma and urine were acceptable, as the correlation coefficient was always adequate (*r*^2^ > 0.990). For low level metabolites, the LOQ was in the range of a few ng/Ml for both matrices, except for some compounds that behaved differently. As an example, 2-AM had a LOQ of 3.9 ng/mL in urine, while in plasma it went up to 78.1 ng/mL. For high level metabolites (BCAAs, TRP, TYR and PHE), it is not necessary to achieve an LOQ of few ng/mL and the value was set as the lowest calibration point falling within the linear range.

The use of Ostro 96-well plates with modifications enabled us to achieve satisfactory recovery, over 85% in plasma and 80% in urine spiked at low concentration. Similarly, accuracy at medium spiked concentration was excellent for both matrices, with CV being below 15%.

Several metabolites saw a decrease in recovery and poorer accuracy at low spiked concentration; this was due to the fact that spiked amounts were close to the LOQ, and analytical error may therefore be greater. The quantification of GABA was significantly deviated in both matrices. This is because GABA was not retained with our experimental setup and eluted with the chromatographic front.

We propose using data on GABA to detect fold changes rather than to provide absolute quantification. For better quantification of most polar metabolites, HILIC may still represent an appropriate method. The presence of co-eluting compounds may affect the ionization of the targeted metabolites producing ME. In this research, ME was negligible for most metabolites, ranging between 80–120% in both plasma and urine. Ion suppression by matrix components significantly affected quantification for VAL, DA, MET, QA and LEU in plasma, together with 2-AM and TYRA in urine. These metabolites are the most polar of those tested and were eluted at the beginning of the chromatographic run, before 2.5 min. Several attempts were made to improve chromatographic separation and MS response. For example, starting with 100% mobile phase A or keeping it isocratically at 95%A for a few minutes increased retention, but the peak shapes got worse, hampering integration and thus quantification. Phospholipids were efficiently removed from plasma by Ostro 96-well plates, while no significant effects were detected in urine.

Removal of matrix contaminant, together with splitting of the UHPLC flow to waste during non-acquisition time, along with good column cleaning, enabled us to acquire up to 300 samples per batch without any significant shift in RT and alteration in the MS response. This is very important in clinical applications, where simultaneous analysis of a large number of samples in the lowest number of separate batches is desirable.

### 3.6. Method Application

The availability of a large number of samples both for plasma (Rhineland Study) and urine (DONALD Study) allowed us to monitor the typical range of presence for each of these metabolites in two independent German populations. The method allowed the quantification of 24 out of 39 metabolites in plasma and 30 metabolites in urine (Table 2). In general, the results were in agreement with published results, despite some biological variation (Table S4).

This information is relevant for the analyst, since it allowed us to verify that the method allows quantitative analysis of 39 out of the 40 target metabolites (all of them except GABA). More detailed analysis of multiple factors of variability influencing the concentration of these compounds is outside the scope of this paper and will be the subject of other publications.

### 3.7. Study Strengths and Limitations

Most of the studies available in the literature cover a limited number of metabolites related to TRP, TYR, BCAAs and gut-derived metabolites [49,50,51]. To achieve a full understanding of microbial metabolite–host interaction in homeostasis and diseases, we validated an analytical method for the separation and detection of 39 metabolites, targeting key branches of different metabolic pathways simultaneously, in particular those related to the microbiota-gut-brain axis [10], covering the different forms of TRP usage, simultaneously investigating serotonergic metabolism [25] and KP metabolism [22].

Our quantitative results suggest that the method is suitable for high-throughput applications in clinical studies, covering an unprecedented number of crucial metabolites in a single analysis. It represents the starting point for future research, and other metabolites of interest can be inserted as required.

The method was designed for low sample requirements, minimal sample handling and working steps, fast extraction, high sample throughput and fast instrumental analysis of 14 min per sample. Up to 384 samples (4 well plates) can be extracted by a single operator per day, and up to 96 samples can be acquired per day.

The method was independently validated on plasma and urine, in order to support multi-compartment studies, allowing direct comparison of metabolite concentrations in both biofluids.

One limitation of this study relates to pre-analytical sample management, from sample collection to handling and storage, which can affect sample quality. This issue was outside the scope of this work, but we are aware it is important for the final results [53,54,55,56,57]. For example, accurate measurement of 5-HT in the whole blood sample is affected by 5-HT instability and reflects platelet 5-HT [58,59,60]; inappropriate blood sample handling can lead to inaccurate results. To avoid these problems, we relied on standard laboratory practices to prepare samples, such as those highlighted in [57]. In this particular case, we selected fasting plasma; EDTA blood was collected and centrifuged within 10 min of collection, and aliquoted and stored at −80 °C within 2 h of collection. EDTA is commonly used as anticoagulants for the generation of platelet-free plasma [61].

Urine was collected as 24-h samples in order to obtain an overall picture of an individual’s metabolic excretion and to eliminate the wide variability observed for spot urine collection. Urine pH can affect the final results; to avoid this problem urine pH was checked at sampling (pH range: 4.9-7.9) and all the samples fell within the desired range [62]. Furthermore, since the samples were stored at low temperature, urine pH was expected to be stable until the analytical phase [62].

The attention of researchers on complex interactions between gut bacteria and human brain has increased in recent years [15,16,22,25]. In 2013, the term “psychobiotics” was introduced to define beneficial bacteria that, when ingested in appropriate quantities (probiotics), exert positive effects in psychiatric patients by influencing the gut bacteria-brain-relationship [63]. This definition was then expanded to prebiotics, food components that support growth of intrinsic commensal bacteria, but this concept could be extended to any substance that “exerts a microbiome-mediated psychological effect” [64]. The psychobiotics treatment could be an interesting strategy to improve life of people suffering from psychiatric disorders but further studies are needed to facilitate its development [64,65]; our validated method can be a complementary tool to evaluate the direct effect of psychobiotics on TRP, TYR, BCAA and gut-derived metabolites.

## 4. Materials and Methods

### 4.1. Reagents and Chemicals

Gamma-aminobutyric acid (HMDB0000112), tryptamine (HMDB0000303) and l-tyrosine (HMDB0000158) were purchased from Fluka (Milan, Italy); 2-aminophenol (ChemSpider ID 5596; PubCHem CID 5801), 3,4-dihydroxyphenyl acetic acid (HMDB0001336), 3,4-dihydroxyphenyl acetic acid-d5, 3-hydroxykynurenine (HMDB0011631), 5-methoxyindole-3-acetic acid (HMDB0004096), 5-methoxytryptamine (HMDB0004095), indole-3-acetic acid (HMDB0000197), indole-3-carboxaldehyde (HMDB0029737), indole-3-carboxylic acid (HMDB0003320), indole-3-lactic acid (HMDB0000671), indole-3-propionic acid (HMDB0002302), indoxyl sulfate (HMDB0000682), kynurenine (HMDB0000684), melatonin (HMDB0001389), dl-phenylalanine (HMDB0000159), picolinic acid (HMDB0002243), tryptophan methyl ester (ChemSpider ID 70366, PubCHem CID 77980) and tyramine (HMDB0000306) were purchased from Sigma (Milan, Italy); 1-acetylisatin (ChemSpider ID 10845, PubCHem CID 11321), 3-hydroxyanthranilic acid (HMDB0001476), 3-methoxy-p-tyramine (HMDB0000022), 5-hydroxyindole-3-acetic acid (HMDB0000763), 5-hydroxyindole-3-acetic acid-d5, 5-hydroxy-tryptophan (HMDB0000472), anthranilic acid (HMDB0001123), dopamine (HMDB0000073), dopamine-d4, homovanillic acid (HMDB0000118), indole-3-acetamide (HMDB0029739), indole-3-acetonitrile (HMDB0006524), kynurenic acid (HMDB0000715), kynurenic acid-d5, l-isoleucine (HMDB0000172), l-leucine (HMDB0000687), l-valine (HMDB0000883), methionine (HMDB0000696), methionine-d4, *N*-acetyl-5-hydroxytryptamine (HMDB0001238), quinolinic acid (HMDB0000232), serotonin (HMDB0000259), serotonin-d4. l-tryptophan (HMDB0000929), l-trypophan-d5, l-tyrosine-d4 and xanthurenic acid (HMDB0000881) were purchased from Spectra 2000 (Rome, Italy). Human citrated plasma was obtained from Sigma (Milan, Italy).

LC-MS grade acetonitrile (ACN), methanol (MeOH) and 2-propanol were purchased from Honeywell (Monza, Italy), LC-MS grade FA was purchased from Sigma (Milan, Italy). Ultrapure Milli-Q deionized water was obtained from Elix (Merck-Millipore, Milan, Italy). OSTRO 96-well plates (25 mg) were purchased from Waters (Milan, Italy). Human plasma was purchased from Sigma Aldrich (Milan, Italy).

### 4.2. Preparation of Stock Solution and Calibration Curves

Stock solutions (1000 mg/mL) were prepared by dissolving each standard in methanol except TYR, TYR-d4 and 3-OH-KYN, which were dissolved in 1 M HCl, KA-d5 in MeOH: DMSO 1:1 (*v*/*v*) and XA in DMSO. The concentration ranges are reported in Table S3.

### 4.3. Method Validation

#### 4.3.1. Linearity and LOQs

Calibration standards were evaluated at 14 concentration levels, prepared with serial dilution in water with 0.1% FA. A linear polynomial model was employed with 1/X weighting factor. The method was considered linear in a specific concentration range if the correlation coefficient (^2^) was equal to or greater than 0.990. The calibration range was designed according to data available in literature, public databases (www.hmdbr.ca) and from analysis of real plasma and urine samples. LOQs were calculated by estimating the calibration points with a signal-to-noise ratio (S/N) of 10.

#### 4.3.2. Recovery, Intra- and Inter-Day Accuracy and Precision, and RT Stability

Analytical recovery was assessed by spiking standards in plasma and urine at low, medium and high concentrations according to the calibration ranges described above. The low and high concentration were set as 5-fold lower or higher than the medium values. As both matrices contained tested metabolites at a different concentration, the spiked concentration was calculated as the % of metabolite recovered compared to the spiked concentration after subtracting the average response from the blank (unspiked) sample. Calibration curves in water with 0.1% FA were used for recovery determination. See Tables S1 and S2 for further details.

Intra-day repeatability was assessed by analysing samples (*n* = 5) spiked at low, medium and high concentration for both plasma and urine within one day. Inter-day repeatability was assessed by analysing QC (*n* = 5), analysed on three separate days (1, 3 and 5). Precision is expressed as the coefficient of the variation percentage (CV%) estimated for spiked QC, after subtracting the concentration of unspiked samples. For acceptance, CV was required to be within 15% at the medium and high concentration and within 20% at the low concentration.

#### 4.3.3. RT Stability

RT stability was assessed by analysis of metabolites detected in 1000 plasma and 672 urine samples. For metabolites not detected in biological samples, the RT was obtained from analysis of QC spiked at medium standard concentration.

#### 4.3.4. Analysis of Blank Samples and ME

Urine blanks (5X) were prepared as described in paragraph 4.4.1 by diluting 25 μL of sample in 225 μL of water with 0.1% FA. Plasma blanks (5X) were prepared as described in paragraph 4.4.1.2 by adding 20 μL of ACN 1% FA to 50 μL plasma instead of standard mix.

MEs were evaluated using a MMC approach. Solvent calibration slopes (SC) were compared with those obtained by fortifying the biological fluids and the deviation was calculated as follows: % of variation = (MMC slope/SC slope) × 100. For plasma, 50 μL of sample and 20 μL of ACN 1% FA (MMC) were loaded into Ostro 96-well plates and extracted as described below. Dried samples were reconstituted in 100 μL of water/ACN (8:2, *v*/*v*), 0.1% FA at appropriate concentration levels. For urine, 25 μL of deionized water (SC) or urine (MMC) were diluted with 225 μL 0.1% FA spiked at appropriate levels.

#### 4.3.5. Carryover Effect and Phospholipid Removal

The carryover effect was assessed by injecting neat ACN after the highest calibration points of SC and MMC and after the acquisition of each recovery batch.

Plasma clean up from phospholipids was addressed by performing a PIS in positive ion mode at *m/z* 184.03 (protonated phosphocholine), which is specific for PC and SM. 50 μL of plasma were directly mixed with 50 μL of water: ACN 8:2 (*v*/*v*), 0.1% FA in order to achieve the same dilution obtained after plasma extraction on Ostro 96-well plates. For urine, the comparison was conducted with undiluted and 10-fold diluted urine.

### 4.4. Extraction Procedures

#### 4.4.1. Urine

Urine was thawed on ice and 25 µl aliquots were loaded into 96-well multifilter plates (Millipore) together with 225 µl of internal standard mix (500 ng/mL) in water with 0.1% FA. 96-well plates were shaken on a vortex for 15 sec, and subsequently filtered using a positive pressure-96 manifold (Waters). Samples were collected in 350 μL 96-well plates and kept at −80 °C until analysis.

##### Plasma

Plasma aliquots (50 μL) were loaded onto an Ostro 96-well plate (Waters, Milan, Italy) and 20 μL of internal standard mix in ACN 1% FA (500 ng/mL) were added. Protein precipitation and metabolite extraction were performed by adding 150 μL of ice-cold ACN 1% FA. Plates were covered, vortexed for 15 sec and placed on an Eppendorf shaker for 10 min at 500 rpm (Eppendorf, Milan, Italy), then filtered using a positive pressure-96 manifold (Waters). The extraction procedure was repeated by adding 150 μL of ice-cold ACN 1% FA. Extracts were brought to dryness with a gentle stream of nitrogen at 37 °C using a Techne Dr-block DB 3D heater, re-dissolved in 100 µl of water: ACN 8:2 0.1 % FA and transferred into 350 μL 96-well plates, kept at −80 °C until analysis.

##### LLE of Urine and Plasma

100 uL of plasma or urine were extracted using 200 uL of ice-cold ACN containing internal standards. The mixtures were first shacked for 30 min at 500 rpm (5 °C). Then, mixtures were stored 1 h at −20 °C to improve protein precipitation and then centrifuged at 17,968 *g* (14000 rpm) for 15 min at 4 °C. Afterwards, the supernatants were collected and stored at −80 °C until analysis. The supernatants were directly injected.

### 4.5. Ultra High Performance Liquid Chromatography-Electrospray Ionization-Triple Quadrupole-Mass Spectrometry (UHPLC-ESI-QqQ-MS)

Detection was performed on a Waters^®^ Xevo TQ MS Triple Quadrupole equipped with ESI source and coupled online with an Aquity UHPLC (Waters, Milford, MA, USA). The MS operated in positive ionization mode, setting the capillary at 270 °C, the source at 300 °C and source voltage at 3 kV. Detection of IS, DOPAC, DOPAC-d5 and HVA were performed in negative ion mode in the same run by setting polarity switching. Ultra-high purity argon was used as collision gas. MS and MS/MS conditions were optimised via software (Intellistart, Waters, Milford, MA, USA) by infusing analytical standards.

#### 4.5.1. RP C_18_ Chromatography

Chromatographic separation was performed using a Water UPLC HSST3 (1.8 μm, 2.1 × 150 mm, 100 A pore diameter) purchased from Waters (Milan, Italy). Mobile phase A was water with 0.1% FA, B was ACN 0.1% FA. The gradient started with 5% B and was maintained for 0.5 min; then % B was increased to 10% at 2.5 min, 15% at 3.5 min, 25% at 4.5 min, 35% at 5.5 min, 45% at 6.5 min, 55% at 7 min and then to 100%B at 7.5 min. Final conditions were retained for 3 min and the column was re-equilibrated to the initial conditions for 4 min. The total run time including column re-equilibration was 14 min. The flow rate was 0.3 mL/min, the injection volume was 2 μL and the column oven was set at 40 °C. The weak and strong solvent washes were water: MeOH (9:1, *v*/*v*) and water: ACN: MeOH: 2-propanol (1:1:1:1, *v/v/v/v*) respectively. Data were acquired and processed with Mass Lynx 4.1 software (Waters).

#### 4.5.2. HILIC Chromatography

A Waters ACQUITY BEH AMIDE 1.7 μm, 2.1 x 150 mm analytical column was used. The mobile phases consisted of (A) 10 mM ammonium formate and 0.2% FA in water: ACN (1:1, *v*/*v*), and (B) 10 mM ammonium formate and 0.2% FA in water: ACN (5:95, *v*/*v*). A multi-step elution gradient was developed as follows, at a flow rate of 0.5 mL/min: at 0.0 min, 100% B a gradient up to 4.0 min, 90% B; then %B was decreased to 70% at 8.0 min, 60% at min 9.0, 50% at min 9.5 and maintained isocratically until min 11.0. Lastly, a reconditioning period up to 1.5 min at 100% B was used. The sample injection volume was 5 μL and the autosampler temperature was kept at 5 °C. The weak and strong solvent washes were water: ACN: MeOH: 2-propanol (1:1:1:1, *v/v/v/v*) and water: MeOH (9:1, *v*/*v*) respectively.

### 4.6. Method Application to Biological Samples

This analysis was carried out as part of the European Joint Programming Initiative, “A Healthy Diet for a Healthy Life” Metabolic HEALTH through Nutrition, Microbiota and Tryptophan bioMARKers (HEALTHMARK) project. The project aims to investigate the complex associations between microbiota and microbiota-derived bioactives of the TRP metabolism, diet and metabolic health. The applicability of the method was assessed by analysis of 24-h urine (*n* = 672, mean age of 16 years with 50.5% males and 49.5% females) collected within the DONALD study and of plasma samples (*n* = 1000) obtained from adults (age ≥ 30 years) collected within the Rhineland study. Data were analysed using Statistica v. 13.3 (TIBCO Software Inc., Palo Alto, CA, USA).

#### 4.6.1. The DONALD Study

The DONALD study is an ongoing, open cohort study conducted in Dortmund, Germany by the Unit of Nutritional Epidemiology, Department of Nutrition and Food Sciences, University of Bonn, Bonn, Germany. This study has collected data on the diet, growth, development and metabolism of apparently healthy children and adolescents since 1985. Children are enrolled at 3 months of age. The collection of 24-h urine samples is part of the annual assessments as soon as the children can provide the samples. The pre-analytical urine pH range was between 4.9 and 7.9, therefore suitable for metabolite quantification [62]. All samples were stored at −22 °C without the addition of preservatives or chemicals and then at −80 °C until laboratory analysis. Further details on the DONALD study [66] and urine collection and storage [67] have been described elsewhere. The DONALD study was approved by the Ethics Committee of the University of Bonn according to the guidelines of the Declaration of Helsinki (approval number 098/06). Written consent was obtained from parents and later on from study participants.

#### 4.6.2. The Rhineland Study

The Rhineland Study is an ongoing community-based cohort study in which all inhabitants of two geographically defined areas in the city of Bonn, Germany aged 30–100 years are being invited to participate. Persons living in these areas are predominantly German with Caucasian ethnicity. Participation in the study is possible by invitation only. The only exclusion criterion is insufficient German language skills to give informed consent. Approval to undertake the study was obtained from the ethics committee of the University of Bonn, Medical Faculty (approval number 338/15). All participants gave written informed consent.

Fasting blood was collected from all participants between 7:00 and 9:30 am, including 2 × 10 mL EDTA blood (EDTA Vacutainer K2), an anticoagulant commonly used for the generation of platelet-free plasma. Plasma is centrifuged for 10 min at 2000× *g* at 20 °C within 10 min of blood collection; centrifuge brake is set on off to avoid platelet activation. Automated aliquoting of the plasma takes place within less than 35 min after centrifugation into 500 μL aliquots (Hamilton Microlab Star). All aliquots are directly cooled (10 °C) during the process and are placed into a chest freezer (−80 °C) within less than 45 min after aliquoting. Time points of blood withdrawal, centrifugation, aliquoting and freezing time are documented in the laboratory information management system (LIMS).

## 5. Conclusions

A UHPLC-ESI-QqQ-MS method was developed for high-throughput, accurate quantification of 39 metabolites related to TRP, TYR, BCAAs and several gut-derived metabolites. The inclusion of a large number of known metabolites, whose concentration is driven by microbial metabolite–host interaction, provided a new metabolomic profiling method suitable for supporting clinical investigation of several important biological questions and opening up new possibilities for nutritional studies aimed at understanding and preventing disease.

Metabolite extraction from plasma was designed on Ostro 96-well plates in order to ensure protein precipitation, lipid removal and good metabolite recovery. For urine, we opted for filtration and 10-fold dilution in order to ensure the simultaneous analysis of metabolites present in different concentration ranges.

The new method was then tested on a large number of real human plasma and urine samples obtained from two main observational and epidemiological studies across the lifespan. This allowed us to estimate the typical working concentration range for each analyte, and to verify that application of the method to real samples representative of Central Europe subpopulations falls within the validation conditions of the method.

## Figures and Tables

**Table 1:** Internal standard used for quantification, RT (min) and MS parameters (parent *m/z*, polarity, quantifier and qualifier ions *m/z*, CV and CE) for the selected analytes. A Waters ACQUITY HSST3 (1.8 µm, 2.1 × 150 mm) column was used for metabolite separation.

Metabolite	Internal Standard	RT (min)	Parent *m/z*	ESI	Q *m/z*	q *m/z*	CV (V)	CE (eV)
γ-aminobutyric acid	MET-d_4_	1.16	104.03	+	68.95	86.14	12	14
l-valine	MET-d_4_	1.47	118.03	+	55.01	72.02	12	18
picolinic acid	MET-d_4_	1.53	124.00	+	77.96	105.87	26	10
dopamine-d_4_		1.66	158.16	+	94.85	122.4	12	22
dopamine	DA-d_4_	1.67	154.22	+	91.02	119.01	12	20
methionine-d_4_		1.68	154.09	+	59.17	62.95	12	16
methionine	MET-d_4_	1.68	150.22	+	104.02	56.04	12	10
2-aminophenol	TRP-d_5_	1.70	110.16	+	92.00	65.01	20	14
quinolinic acid	MET-d_4_	1.80	168.22	+	77.98	106.03	14	16
3-hydroxykynurenine	TRP-d_5_	2.01	225.176	+	110.02	162.01	14	18
tyrosine-d_4_		2.04	186.16	+	140.11	93.95	12	14
tyrosine	TYR-d_4_	2.07	182.17	+	136.07	90.96	18	16
l-isoleucine	MET-d_4_	2.25	132.09	+	86.00	69.00	10	12
tyramine	TYR-d_4_	2.25	138.12	+	76.68	103.97	10	24
l-leucine	MET-d_4_	2.38	132.09	+	86.00	43.00	10	12
serotonin-d_4_		2.93	181.16	+	118.14	146.05	12	26
serotonin	5-HT-d_4_	3.02	177.22	+	115.09	132.18	10	26
5-hydroxy-tryptophan	TRP-d_5_	3.00	221.29	+	162.01	134.02	12	18
3-methoxy-*p*-tyramine	TYR-d_4_	3.02	168.22	+	91.00	119.05	8	20
kynurenine	TRP-d_5_	3.53	209.12	+	94.01	146.08	14	16
dl-phenylalanine	TYR-d_4_	3.61	166.22	+	120.10	103.01	14	20
3-hydroxyanthranilic acid	TRP-d_5_	4.75	154.22	+	80.01	108.01	10	22
tryptophan-d_5_		4.90	210.16	+	150.09	122.11	12	18
tryptophan	TRP-d_5_	4.94	205.29	+	146.06	118.01	12	16
1-acetylisatin	TRP-d_5_	4.94	190.01	+	148.01	162.01	18	10
DOPAC-d_5_		4.99	172.11	-	128.04	99.99	14	8
3,4-dihydroxyphenyl acetic acid	DOPAC-d_5_	5.04	167.07	-	123.05	94.99	14	8
xanthurenic acid	TRP-d_5_	5.03	206.09	+	160.00	132.02	20	18
kynurenic acid-d_5_		5.41	195.09	+	149.06	121.08	24	18
kynurenic acid	KA-d_5_	5.44	190.09	+	143.99	116.00	20	20
tryptamine	TRP-d_5_	5.45	161.13	+	127.20	117.40	12	24
5-methoxytryptamine	TRP-d_5_	5.60	191.20	+	159.09	143.08	12	22
5-hydroxyindole acetic acid-d_5_		5.71	197.16	+	150.16	122.17	16	14
5-hydroxyindole acetic acid	5-OH-IAA- d_5_	5.74	192.23	+	146.27	91.00	18	14
*N*-acetyl-5-hydroxytryptamine	TRP-d_5_	5.86	219.20	+	160.07	115.09	16	16
tryptophan methyl ester	TRP-d_5_	6.07	219.14	+	160.00	132.02	12	18
homovanillic acid	DOPAC-d_5_	6.20	181.09	-	137.08	121.99	8	10
indoxyl sulfate	TRP-d_5_	6.24	212.04	-	80.08	132.02	24	20
indole-3-acetamide	TRP-d_5_	6.53	175.05	+	102.99	76.95	14	30
anthranilic acid	TRP-d_5_	6.78	138.22	+	91.99	65.04	10	22
indole-3-lactic acid	TRP-d_5_	6.96	206.11	+	160.09	130.02	18	10
indole-3-carboxylic acid	TRP-d_5_	7.15	162.08	+	116.03	88.95	16	20
melatonin	TRP-d_5_	7.31	233.22	+	174.08	159.05	16	14
5-methoxyindole acetic acid	TRP-d_5_	7.35	206.17	+	160.17	145.05	16	16
indole-3-carboxaldehyde	TRP-d_5_	7.36	146.09	+	118.05	90.97	22	24
indole-3-acetonitrile	TRP-d_5_	7.52	130.22	+	76.95	102.99	30	22
indole-3-acetic acid	TRP-d_5_	7.53	176.09	+	130.00	102.99	18	12
indole-3-propionic acid	TRP-d_5_	8.06	190.11	+	130.02	54.96	12	16

**Table 2:** Metabolite concentration ranges (μM) detected in plasma and urine. n.d.: not detected; n.a.: data not available.

	Plasma (μM)			Urine (μM)		
Metabolite	Min	Median	Max	Min	Median	Max
l-valine	12.09	62.12	130	1.678	29.15	94.71
picolinic acid	0.00179	0.0198	0.057	0.649	1.402	2.488
dopamine	0	0.0128	0.0718	0.29	2.089	8.029
methionine	3.09	11.45	25.10	0	2.158	36.23
2-aminophenol	n.d.			n.d.		
quinolinic acid	0.414	1.404	9.694	10.58	40.16	146
3-hydroxykynurenine	n.d.			0	0.357	3.870
tyrosine	6.721	27.86	71.30	6.013	136	849
l-isoleucine	4.924	26.46	77.95	0.106	12.69	55.82
tyramine	n.d.			0.197	4.518	139
l-leucine	10.71	57.07	120.0	1.763	33.05	158
serotonin	0	0.167	1.047	0.04	0.492	1.905
5-hydroxy-tryptophan	n.d.			0.0394	0.151	0.723
3-methoxy-*p*-tyramine	n.d.			0.0846	0.346	1.722
kynurenine	0.450	1.270	3.479	0.215	3.703	43.88
dl-phenylalanine	8.096	27.86	71.30	2.342	18.08	107
3-hydroxyanthranilic acid	0.177	0.203	0.322	0.0808	0.412	4.494
tryptophan	8.499	29.82	81.49	7.051	64.02	366
1-acetylisatin	n.d.			n.d.		
3,4-dihydroxyphenyl acetic acid	0	0.0477	73.7	n.d.		
xanthurenic acid	0.02	0.0661	0.183	0.561	5.424	36.10
kynurenic acid	0.00553	0.0185	0.167	3.334	20.38	91.75
tryptamine	n.d.			0.0272	0.449	2.467
5-methoxytryptamine	n.d.			n.d.		
5-hydroxyindole acetic acid	0.0164	0.0447	0.456	0.0268	19.68	89.54
*N*-acetyl-5-hydroxytryptamine	n.d.			n.d.		
tryptophan methyl ester	n.d.			n.d.		
homovanillic acid	0.0118	0.0782	1.0	9.313	35.58	136
indoxyl sulfate	0.0491	2.744	12.99	n.a.		
indole-3-acetamide	n.d.			0.0170	0.272	10.07
anthranilic acid	n.d.			0.0950	0.401	2.058
indole-3-lactic acid	0.0759	0.697	4.009	0.198	1.165	18.90
indole-3-carboxylic acid	n.d.			0.0305	0.0994	7.279
melatonin	n.d.			n.d.		
5-methoxyindole acetic acid	n.d.			n.d.		
indole-3-carboxaldehyde	0.0103	0.0494	0.186	0.00245	0.123	3.992
indole-3-acetonitrile	0.326	2.003	31.72	3.116	15.60	96.82
indole-3-acetic acid	0.292	1.51	23.01	6.114	30.09	205
indole-3-propionic acid	0	1.156	12.75	0.0187	0.0557	2.197

**Table 3:** Recovery obtained from analysis of 21 pre-selected metabolites using LLE or Ostro 96-well plate. The selected metabolites were: GABA, 2-AM, VAL, MET, ILE, LEU, DA, 5-HT, PHE, TYR, 3-ME-TYRA, TRP, AA, 1-acetylisatin, 3-OH-AA, IACN, IAA, KA, XA, NA-5-HT, 5-OH-TRP and alpha-chloralose.

Recovery (Average = 5)		Extraction Methods
21 pre-selected metabolites	LLE	Ostro 96-Well plate
<50	1	1
50–60	1	1
60–70	5	0
70–80	3	1
80–90	4	1
90–100	3	15
>100	4	2

## Supplementary Materials

The following are available online at https://www.mdpi.com/2218-1989/9/11/261/s1. Table S1: Calibration curve range of linearity, coefficient of correlation, limit of quantification, LOQ, matrix effect and retention time stability in plasma and urine; Table S2: Recovery and precision at low, medium and high spiked concentrations in plasma and urine; Table S3: stock solution and spiked concentrations (low, medium and high) used for recovery and precision assays in plasma and urine. Table S4: Metabolite concentration ranges detected in plasma and urine compared to reference data. Figure S1: Chromatogram of 13 standard compounds obtained with RP and HILIC chromatography. Figure S2: Chromatogram of the most polar metabolites obtained on five different C_18_ analytical columns.
