# Supplementary material for: Metabolic Profiling of Human Plasma and Urine, Targeting Tryptophan, Tyrosine and Branched Chain Amino Acid Pathways
Source: Metabolites. 2019 Nov 1;9(11):261. doi: 10.3390/metabo9110261 (PMC6918267; doi:10.3390/metabo9110261)
Supplement: Supplementary file 1 [file metabolites-09-00261-s001.pdf]

Paper Article

Metabolic Profiling of Human Plasma and Urine, Targeting Tryptophan, Tyrosine and Branched Chain Amino Acid Pathways

Andrea Anesi [1,°], Josep Rubert [2,°], Kolade Oluwagbemigun [3], Ximena Orozco-Ruiz [4], Ute Nöthlings [3], Monique M.B. Breteler [4,5] and Fulvio Mattivi [2,6*]

[1] Department of Food Quality and Nutrition, Research and Innovation Centre, Fondazione Edmund Mach (FEM), Via E. Mach 1, 38010 San Michele all' Adige, TN, Italy
[2] CIBIO, Department of Cellular, Computational and Integrative Biology, Via Sommarive 9, 38123, Povo, TN, Italy
[3] Institute of Nutrition and Food Sciences, Nutritional Epidemiology, Rheinische Friedrich-Wilhelms-Universität Bonn, Endenicher Allee 19b, 53115 Bonn, Germany
[4] Population Health Sciences, German Center for Neurodegenerative diseases (DZNE), Bonn, Germany. Venusbergcampus 1 - Building 99, 53127 Bonn, Germany
[5] Institute for Medical Biometry, Informatics and Epidemiology (IMBIE), Faculty of Medicine, University of Bonn, Venusberg-Campus 1 - Building 11, 53127 Bonn, Germany
[6] University of Trento, Department of Physics, Bioorganic Chemistry Laboratory, Via Sommarive 14, 38123 Povo, TN, Italy
° Authors contributed equally to the manuscript

* Correspondence: fulvio.mattivi@unitn.it; Tel.: +39-0461-615-259 (F.M.)

SUPPLEMENTARY MATERIAL

Table S1. Calibration curve range of linearity (ng/mL), coefficient of correlation (r^2), LOQ (ng/ml), matrix effect (%) and retention time stability (CV%) in plasma and urine

Metabolite	Range (ng/mL)		r^2		LOQ (ng/ml)		Matrix Effect		RT (CV%)	
	Plasma	Urine	Plasma	Urine	Plasma	Urine	Plasma	Urine	Plasma	Urine
**γ-aminobutyric acid**	1.2-1250	6.1-3125	0.991	0.995	<6.1	1.2	2.4	19.3	0.65	0.38
**L-valine**	24.4-6250	19.53-5000	0.990	0.992	<19.5	<24.4	55.5	69.5	0.70	0.51
**picolinic acid**	0.9-125	1.9-62.5	0.994	0.998	1.9	1.9	84.0	103.7	0.69	0.59
**dopamine-d_4**	19.5-5000	2.4-5000	0.993	0.998	<4.8	<19.5	54.0	36.1	0.63	0.29
**dopamine**	0.4-500	0.4-500	0.993	0.998	0.9	0.4	52.9	35.4	0.63	0.30
**methionine-d_4**	4.8-2500	19.5-5000	0.990	0.993	<19.5	4.8	51.5	47.6	0.63	0.30
**methionine**	19.5-5000	2.4-1250	0.992	0.993	<2.4	<19.5	52.4	43.9	0.63	0.32
**2-aminophenol**	78.1-2500	3.9-250	0.994	0.995	3.9	<78.1	83.5	54.5	2.10	0.18
**quinolinic acid**	39.0-2500	24.4-6400	0.992	0.998	<24.4	39.0	49.3	80.7	0.63	0.27
**3-hydroxykynurenine**	7.8-500	15.6-25600	0.991	0.997	15.6	<7.8	94.1	101.1	0.63	0.59
**tyrosine-d_4**	39.0-10000	9.7-2500	0.996	0.995	<9.7	<39.0	100.8	95.0	1.04	0.44
**tyrosine**	12.2-12500	12.2-6250	0.996	0.998	24.4	<24.4	112.2	108.4	0.51	0.46
**L-isoleucine**	195.3-12500	39.0-5000	0.999	0.999	<39.0	<195.3	91.4	118.9	0.47	0.38
**tyramine**	2.4-2500	9.7-5000	0.998	0.990	<9.7	2.4	116.8	45.6	0.47	0.50
**L-leucine**	97.6-12500	39.0-12500	0.999	0.992	<39.0	<97.6	90.8	81.1	0.43	0.41
**serotonin-d_4**	9.7-5000	9.7-5000	0.994	0.995	<9.7	9.76	95.5	83.2	0.35	0.43
**serotonin**	0.9-250	1.9-250	0.998	0.993	<3.9	<1.9	92.9	83.8	0.34	0.48
**5-hydroxy-tryptophan**	39.0-2500	1.9-250	0.996	0.996	7.8	39.0	90.7	82.9	0.51	0.99
**3-methoxy-*p*-tyramine**	0.9-250	0.9-250	0.991	0.994	<0.9	<1.9	93.8	80.8	0.57	0.44
**kynurenine**	2.4-10000	2.4-10000	0.998	0.995	<2.4	<2.4	91.4	91.8	0.30	0.33
**DL-phenylalanine**	12.2-12500	12.2-6250	0.991	0.999	<12.2	<12.2	83.5	82.7	0.00	0.33
**3-hydroxyanthranilic acid**	31.2-500	3.9-1000	0.992	0.993	3.9	<31.2	96.7	87.4	0.22	0.28
**tryptophan-d_5**	4.8-5000	4.8-5000	0.995	0.993	<4.8	<4.8	90.8	90.2	0.00	0.23
**tryptophan**	24.4-12500	48.8-12500	0.997	0.992	<48.8	<24.4	87.0	83.8	0.00	0.22
**1-acetylisatin**	3.9-250	0.9-125	0.992	0.994	0.9	3.9	99.2	84.1	0.17	0.20
**3,4-dihydroxyphenyl acetic acid -d_5**	156.2-10000	31.2-5000	0.996	0.992	<31.2	156.1	94.9	85.8	0.15	0.19
**3,4-dihydroxyphenyl acetic acid**	156.2-5000	31.2-5000	0.996	0.997	<31.2	156.1	87.0	82.0	0.24	0.24
**xanthurenic acid**	1.9-500	0.6-2500	0.991	0.999	<1.2	<1.9	96.3	80.9	0.21	0.22
**kynurenic acid-d_5**	1.2-5000	2.4-5000	0.999	0.999	<2.4	<1.2	92.9	99.6	0.00	0.21
**kynurenic acid**	0.1-5000	2.4-10000	0.995	0.997	<2.4	0.1	96.0	95.7	0.19	0.20
**tryptamine**	1.9-500	1.9-250	0.992	0.993	1.9	1.9	93.3	97.2	0.26	0.18
**5-methoxytryptamine**	0.9-500	0.9-250	0.998	0.998	<0.9	1.9	98.1	91.3	0.14	0.15
**5-hydroxyindole acetic acid-d_5**	9.7-5000	19.5-5000	0.998	0.996	<19.5	<9.7	92.6	95.4	0.18	0.16
**5-hydroxyindole acetic acid**	1.9-250	3.9-5000	0.998	0.997	3.9	3.9	98.1	89.9	0.18	0.15
**N-acetyl-5-hydroxytryptamine**	0.4-500	0.4-250	0.998	0.998	<0.9	0.9	109.4	104.3	0.13	0.28
**tryptophan methyl ester**	0.9-500	1.9-250	0.999	0.999	1.9	1.9	95.2	105.0	0.15	0.76
**homovanillic acid**	156.2-20000	312.5-10000	0.996	0.994	312.5	156.2	88.9	83.8	0.59	0.16
**indoxyl sulfate**	78.1-12500	78.1-12500	0.998	0.999	<78.1	78.1	100.6	94.9	0.23	1.09
**indole-3-acetamide**	2,4-500	1.9-1000	0.995	0.993	<1.9	2.4	102.2	101.0	0.14	0.17
**anthranilic acid**	0.4-500	0.9-250	0.999	0.998	1.9	1.9	96.2	102.8	0.11	0.15
**indole-3-lactic acid**	1.9-2000	4.8-2500	0.995	0.997	<9.7	<7.8	97.8	102.5	0.15	0.13
**indole-3-carboxylic acid**	0.488-500	1.95-1000	0.998	0.995	<1.95	<0.4	85.3	107.5	0.14	0.21
**melatonin**	0.122-500	0.122-250	0.997	0.999	0.244	0.1	93.0	101.6	0.13	0.14
**5-methoxyindole acetic acid**	0.244-500	0.98-250	0.998	0.998	1.95	0.4	95.6	107.5	0.12	0.15
**indole-3-carboxaldehyde**	0.244-500	0.24-250	0.999	0.999	0.48	<0.9	91.1	93.4	0.14	0.12
**indole-3-acetonitrile**	2.441-5000	4.88-5000	0.995	0.996	<19.53	<4.8	83.6	104.9	0.14	0.11
**indole-3-acetic acid**	1.221-5000	4.88-10000	0.995	0.994	<9.77	2.4	86.9	105.0	0.00	0.10
**indole-3-propionic acid**	1.221-5000	0.48-250	0.995	0.999	<0.48	<1.2	88.5	114.4	0.13	0.12

Table S2. Recovery and precision at low, medium and high spiked concentrations in plasma and urine. Recovery is expressed as the % of metabolite recovered respect to the spiked concentration after subtracting the average response from the blank. Precision is expressed as the coefficient of variation percentage (CV%) estimated for spiked QC, after subtracting concentration of blanks. Intra-day precision was calculated by the analysis of 5 samples on three separate days (DAY 1, 3 and 5) and CV% is reported in brackets. Inter-day (I.D.) repeatability is expressed as the coefficient of variation (CV%).

Metabolite	Recovery % Plasma			Recovery % Urine				Precision (CV%) Plasma			Precision (CV%) Urine		
	LOW	MED	HIGH	LOW	MED	HIGH		LOW	MED	HIGH	LOW	MED	HIGH
**γ-aminobutyric acid**	71.2	90.7	68.4	108.3	81.9	12.8	DAY 1<br>DAY 3<br>DAY 5<br>I.D.	n.d.	n.d.	n.d.	n.d.	n.d.	n.d.
**L-valine**	107.5	80.7	90.8	112.1	86.9	80.0	DAY 1<br>DAY 3<br>DAY 5<br>I.D.	74.3 (0.7)<br>108.0 (0.4)<br>99.8 (0.2)<br>0.4	109.9 (1.2)<br>97.5 (0.7)<br>100.5 (6.7)<br>2.9	30.3 (5.5)<br>57.6 (1.6)<br>57.4 (5.9)<br>4.4	106.1 (1.0)<br>94.9 (0.4)<br>88.1 (2.5)<br>1.3	102.3 (0.7)<br>102.1 (1.2)<br>115.9 (2.6)<br>1.5	60.4 (1.7)<br>77.5 (2.9)<br>88.8 (0.1)<br>1.6
**picolinic acid**	91.0	82.8	71.5	90.7	102.4	102.8	DAY 1<br>DAY 3<br>DAY 5<br>I.D.	89.5 (18.8)<br>101.1 (7.7)<br>97.9 (22.3)<br>16.3	100.9 (5.4)<br>97.7 (3.8)<br>99.1 (0.4)<br>3.2	103.1 (4.9)<br>100.0 (3.9)<br>93.7 (3.6)<br>4.1	98.4 (9.8)<br>105.8 (11.0)<br>98.3 (21.2)<br>14.0	86.5 (4.5)<br>92.8 (14.4)<br>107.7 (3.4)<br>8.2	101.1 (3.1)<br>101.8 (1.7)<br>101.4 (2.2)<br>2.3
**dopamine-d_4**	100.2	95.4	89.2	70.1	107.0	118.6	DAY 1<br>DAY 3<br>DAY 5<br>I.D.	-<br>-<br>-<br>-	96.7 (3.4)<br>93.5 (3.1)<br>82.1 (6.8)<br>4.4	79.0 (4.2)<br>78.0 (1.8)<br>78.3 (2.2)<br>2.7	-<br>-<br>-<br>-	88.0 (5.5)<br>89.2 (5.1)<br>109.5 (5.3)<br>5.3	102.5 (1.3)<br>88.9 (1.7)<br>94.3 (2.8)<br>1.9
**dopamine**	101.5	92.6	85.12	72.5	86.4	91.3	DAY 1<br>DAY 3<br>DAY 5<br>I.D.	-<br>-<br>-<br>-	89.8 (10.5)<br>80.9 (3.8)<br>86.8 (9.4)<br>7.9	75.9 (2.8)<br>61.4 (2.5)<br>63.3 (2.6)<br>2.6	75.9 (4.4)<br>91.0 (1.4)<br>91.2 (6.4)<br>4.1	103.1 (1.9)<br>111.3 (1.3)<br>100.2 (10.9)<br>4.7	85.3 (1.8)<br>78.5 (1.8)<br>89.0 (0.7)<br>1.4
**methionine-d_4**	108.4	111.6	84.5	95.2	81.0	104.0	DAY 1<br>DAY 3<br>DAY 5<br>I.D.	-<br>-<br>-<br>-	99.4 (2.8)<br>103.4 (0.9)<br>101.0 (3.9)<br>2.6	98.4 (1.8)<br>95.2 (0.4)<br>86.5 (1.8)<br>1.3	-<br>-<br>-<br>-	97.8 (5.4)<br>99.9 (10.3)<br>102.1 (2.1)<br>6.0	92.0 (0.3)<br>100.1 (1.5)<br>100.8 (3.6)<br>1.8
**methionine**	104.2	104.3	98.8	106.0	94.0	84.2	DAY 1<br>DAY 3<br>DAY 5<br>I.D.	104.5 (3.5)<br>85.6 (1.8)<br>106.8 (1.7)<br>2.3	91.2 (2.2)<br>90.2 (0.7)<br>93.1 (5.5)<br>2.8	78.9 (2.6)<br>76.8 (1.6)<br>82.1 (4.3)<br>2.8	104.0 (5.8)<br>106.0 (2.7)<br>99.0 (4.3)<br>4.3	99.6 (2.4)<br>103.9 (1.9)<br>106.3 (1.1)<br>1.8	89.9 (0.8)<br>101.9 (0.2)<br>101.2 (0.6)<br>0.5
**2-aminophenol**	91.7	95.3	83.8	161.2	82.1	80.1	DAY 1<br>DAY 3<br>DAY 5<br>I.D.	-<br>-<br>-<br>-	38.5 (10.9)<br>55.7 (5.3)<br>35.9 (25.2)<br>13.8	72.0 (5.9)<br>76.0 (4.1)<br>67.5 (6.1)<br>5.4	-<br>-<br>-<br>-	126.2 (1.9)<br>118.7 (19.6)<br>125.5 (2.1)<br>7.9	93.6 (4.9)<br>103.6 (2.0)<br>110.9 (3.0)<br>3.3
**quinolinic acid**	76.5	103.4	110.1	82.8	95.3	85.9	DAY 1<br>DAY 3<br>DAY 5<br>I.D.	104.1 (10.0)<br>83.9 (3.8)<br>103.8 (5.3)<br>6.4	88.7 (1.6)<br>97.9 (1.4)<br>89.3 (2.4)<br>1.8	109.6 (4.7)<br>102.4 (1.5)<br>116.8 (6.6)<br>4.3	87.6 (0.6)<br>105.6 (0.5)<br>95.0 (1.0)<br>0.7	111.2 (0.5)<br>111.9 (2.0)<br>114.4 (6.4)<br>3.0	96.2 (4.3)<br>103.7 (0.5)<br>111.5 (1.9)<br>2.2
**3-hydroxykynurenine**	97.9	118.5	98.8	70.8	96.2	102.8	DAY 1<br>DAY 3<br>DAY 5<br>I.D.	78.5 (45.0)<br>70.2 (7.7)<br>112.8 (42.5)<br>31.7	103.0 (6.2)<br>100.4 (2.7)<br>96.4 (7.1)<br>5.3	98.4 (4.9)<br>100.7 (3.8)<br>91.0 (11.9)<br>6.9	188.8 (9.8)<br>158.0 (9.1)<br>102.6 (17.1)<br>12.0	102.6 (7.7)<br>117.7 (5.3)<br>106.3 (11.6)<br>8.2	79.6 (2.2)<br>84.1 (6.3)<br>103.2 (5.7)<br>4.8
**tyrosine-d_4**	114.5	108.0	119.7	110.1	101.3	95.8	DAY 1<br>DAY 3<br>DAY 5<br>I.D.	-<br>-<br>-<br>-	108.7 (2.5)<br>107.5 (1.3)<br>101.7 (4.6)<br>2.8	100.0 (2.1)<br>101.1 (0.8)<br>97.8 (1.3)<br>1.4	-<br>-<br>-<br>-	103.2 (0.7)<br>104.3 (0.9)<br>112.0 (3.4)<br>1.6	76.0 (4.1)<br>101.4 (0.2)<br>102.7 (1.7)<br>2.0
**tyrosine**	115.1	98.8	100.6	102.6	83.6	91.5	DAY 1<br>DAY 3<br>DAY 5<br>I.D.	115.4 (1.0)<br>118.5 (0.3)<br>103.5 (4.2)<br>1.8	111.2 (1.0)<br>104.9 (1.0)<br>106.4 (0.4)<br>0.8	98.7 (1.9)<br>99.4 (0.2)<br>89.1 (9.7)<br>3.9	99.6 (0.6)<br>99.7 (1.3)<br>76.5 (2.1)<br>1.3	95.9 (0.7)<br>101.0 (0.5)<br>82.4 (1.1)<br>0.8	36.1 (2.5)<br>37.2 (1.6)<br>52.8 (1.8)<br>1.9
**L-isoleucine**	96.2	100.1	85.1	45.9	82.8	80.2	DAY 1<br>DAY 3<br>DAY 5<br>I.D.	141.4 (0.5)<br>107.2 (1.4)<br>122.1 (0.9)<br>1.0	102.8 (1.0)<br>103.2 (0.6)<br>102.1 (1.0)<br>0.9	89.8 (4.3)<br>98.3 (0.5)<br>80.9 (9.2)<br>4.7	105.0 (1.1)<br>126.9 (1.7)<br>74.1 (9.5)<br>4.1	101.9 (3.1)<br>104.7 (5.4)<br>74.2 (0.7)<br>3.1	81.3 (2.5)<br>100.8 (0.9)<br>100.9 (0.5)<br>1.0
**tyramine**	123.2	100.2	79.9	119.6	100.2	79.9	DAY 1<br>DAY 3	111.5 (10.4)<br>118.1 (11.8)	105.2 (3.7)<br>108.7 (2.5)	95.2 (3.1)<br>101.0 (0.8)	87.3 4.1<br>76.6 (0.4)	92.8 (2.2)<br>93.4 (1.0)	69.8 (1.8)<br>74.6 (0.5)

							DAY 5<br>I.D.	92.6 (5.8)<br>9.3	102.9 (4.0)<br>3.4	99.3 (2.5)<br>2.2	119.8 (1.9)<br>2.2	99.7 (3.8)<br>2.3	100.3 (2.6)<br>1.6
**L-leucine**	89.1	82.0	108.7	77.2	95.7	95.8	DAY 1<br>DAY 3<br>DAY 5<br>I.D.	119.4 (1.4)<br>110.7 (0.0)<br>106.6 (0.7)<br>0.7	99.1 (0.7)<br>114.8 (0.3)<br>98.0 (0.3)<br>0.5	93.0 (3.5)<br>98.6 (0.5)<br>76.1 (10.5)<br>4.7	116.1 (0.9)<br>89.1 (1.2)<br>93.5 (0.7)<br>0.9	106.5 (1.9)<br>111.3 (5.9)<br>113.9 (6.5)<br>4.8	78.3 (1.0)<br>96.9 (0.8)<br>99.0 (0.7)<br>0.9
**serotonin-d_4**	112.9	116.3	90.9	80.5	81.2	80.9	DAY 1<br>DAY 3<br>DAY 5<br>I.D.	-<br>-<br>-<br>-	97.6 (3.0)<br>103.4 (3.8)<br>100.6 (1.6)<br>2.8	95.6 (2.6)<br>100.2 (1.8)<br>96.9 (1.3)<br>1.9	-<br>-<br>-<br>-	103.0 (3.2)<br>94.4 (2.8)<br>106.2 (1.9)<br>2.7	88.3 (1.2)<br>96.9 (1.5)<br>101.5 (1.7)<br>1.5
**serotonin**	98.6	97.9	80.6	73.0	83.5	84.3	DAY 1<br>DAY 3<br>DAY 5<br>I.D.	61.3 (15.3)<br>107.5 (14.2)<br>79.9 (7.0)<br>12.2	101.3 (4.4)<br>113.2 (6.4)<br>98.1 (5.1)<br>5.2	97.0 (2.8)<br>99.5 (0.3)<br>94.1 (5.0)<br>2.7	102.7 (1.3)<br>100.1 (2.0)<br>89.3 (9.1)<br>4.1	112.6 (2.2)<br>103.6 (1.6)<br>116.8 (0.3)<br>1.4	87.9 (0.8)<br>82.5 (2.3)<br>101.6 (1.5)<br>1.6
**5-hydroxy-tryptophan**	84.9	90.4	90.2	99.0	107.6	89.6	DAY 1<br>DAY 3<br>DAY 5<br>I.D.	-<br>-<br>-<br>-	98.2 (2.7)<br>103.3 (16.3)<br>87.7 (5.9)<br>8.3	97.3 (7.6)<br>98.2 (2.7)<br>94.8 (7.5)<br>5.9	177.1 (17.8)<br>182.8 (3.9)<br>179.8 (2.1)<br>7.9	68.2 (4.2)<br>110.9 (12.0)<br>112.0 (1.7)<br>6.0	87.0 (3.9)<br>100.8 (3.8)<br>102.5 (0.6)<br>2.8
**3-methoxy-*p*-tyramine**	85.8	97.8	81.5	89.9	102.7	98.1	DAY 1<br>DAY 3<br>DAY 5<br>I.D.	70.3 (22.9)<br>86.2 (12.7)<br>92.1 (5.0)<br>13.5	100.4 (1.1)<br>111.8 (3.1)<br>96.6 (4.6)<br>2.9	97.5 (4.0)<br>103.3 (1.1)<br>95.8 (5.9)<br>3.7	103.2 (2.3)<br>103.5 (3.4)<br>103.3 (2.2)<br>2.7	103.0 (5.9)<br>103.8 (3.0)<br>111.9 (3.1)<br>4.0	86.3 (1.7)<br>92.5 (2.0)<br>101.6 (0.8)<br>1.5
**kynurenine**	118.3	102.8	98.4	93.5	93.8	92.0	DAY 1<br>DAY 3<br>DAY 5<br>I.D.	101.2 (2.4)<br>93.4 (1.5)<br>93.8 (16.0)<br>6.6	97.3 (3.3)<br>99.8 (1.8)<br>88.1 (14.1)<br>6.4	93.8 (2.5)<br>99.2 (0.6)<br>83.9 (7.8)<br>3.6	113.0 (1.3)<br>97.1 (2.5)<br>68.4 (8.4)<br>4.1	111.6 (0.4)<br>102.1 (3.8)<br>102.5 (4.4)<br>2.9	80.6 1.5<br>94.3 (1.0)<br>100.4 (0.7)<br>1.0
**DL-phenylalanine**	107.7	103.8	98.1	91.6	84.8	89.6	DAY 1<br>DAY 3<br>DAY 5<br>I.D.	98.4 (1.4)<br>93.7 (0.3)<br>96.7 (0.8)<br>0.9	104.2 (1.2)<br>106.8 (0.4)<br>96.6 (0.5)<br>0.7	95.2 (1.9)<br>100.4 (1.8)<br>90.1 (0.3)<br>1.4	110.5 (1.6)<br>69.8 (2.0)<br>96.9 (4.8)<br>2.8	91.4 (1.3)<br>99.1 (3.0)<br>72.2 (0.1)<br>1.5	53.4 (0.9)<br>42.4 (0.6)<br>52.6 (0.8)<br>0.8
**3-hydroxyanthranilic acid**	89.3	92.9	99.8	87.9	86.9	73.9	DAY 1<br>DAY 3<br>DAY 5<br>I.D.	73.7 (22.7)<br>92.6 (7.8)<br>72.4 (25.3)<br>18.6	80.7 (8.2)<br>83.7 (3.3)<br>75.4 (5.5)<br>5.7	97.9 (1.8)<br>101.0 (1.7)<br>91.6 (3.4)<br>2.3	104.2 (6.1)<br>101.8 (8.5)<br>98.0 (8.0)<br>7.5	99.3 (1.9)<br>106.5 (2.6)<br>98.0 (1.7)<br>2.0	50.3 (3.7)<br>52.2 (3.7)<br>49.9 (6.8)<br>4.7
**tryptophan-d_5**	85.3	90.5	81.0	50.5	80.1	80.3	DAY 1<br>DAY 3<br>DAY 5<br>I.D.	-<br>-<br>-<br>-	98.1 (2.8)<br>102.2 (1.8)<br>92.0 (2.2)<br>2.3	94.4 (2.0)<br>97.8 (0.9)<br>85.9 (0.8)<br>1.2	-<br>-<br>-<br>-	103.8 (2.8)<br>99.5 (3.6)<br>99.7 (1.5)<br>2.6	82.1 (2.0)<br>98.9 (1.0)<br>101.6 (2.4)<br>1.8
**tryptophan**	95.2	96.9	82.5	88.2	86.9	108.7	DAY 1<br>DAY 3<br>DAY 5<br>I.D.	111.1 (1.1)<br>111.5 (1.8)<br>103.7 (1.3)<br>1.4	101.6 (0.5)<br>103.8 (0.5)<br>100.8 (5.4)<br>2.1	93.7 (1.7)<br>100.4 (0.5)<br>93.2 (5.8)<br>2.7	113.5 (0.6)<br>115.3 (0.6)<br>114.9 (4.2)<br>1.8	91.4 (0.7)<br>108.1 (3.1)<br>90.4 (0.8)<br>1.5	42.2 (0.9)<br>49.0 (1.1)<br>59.9 (1.5)<br>1.1
**1-acetylisatin**	100.2	100.8	97.2	92.8	110.3	99.0	DAY 1<br>DAY 3<br>DAY 5<br>I.D.	96.8 (12.7)<br>116.1 (7.4)<br>89.6 (9.7)<br>9.8	108.9 (26.4)<br>106.5 (3.2)<br>89.9 (10.2)<br>13.2	92.5 (8.7)<br>102.1 (5.8)<br>87.1 (3.8)<br>6.1	129.8 19.8<br>133.3 (1.9)<br>124.3 (2.8)<br>8.2	100.9 (6.8)<br>100.7 (2.5)<br>111.1 (1.5)<br>3.6	83.3 (3.9)<br>97.3 (9.5)<br>97.8 (3.6)<br>5.7
**3,4-dihydroxyphenyl acetic acid-d_5**	86.4	104.3	100.2	103.5	101.4	100.5	DAY 1<br>DAY 3<br>DAY 5<br>I.D.	-<br>-<br>-<br>-	93.6 (2.7)<br>100.7 (0.4)<br>96.3 (5.9)<br>3.0	99.5 (3.6)<br>103.0 (2.9)<br>101.0 (1.2)<br>2.5	-<br>-<br>-<br>-	103.7 (0.7)<br>107.9 (5.5)<br>100.0 (1.8)<br>2.7	97.6 (2.0)<br>100.5 (1.9)<br>101.5 (0.2)<br>1.4
**3,4-dihydroxyphenyl acetic acid**	87.0	83.4	97.8	97.8	98.8	96.9	DAY 1<br>DAY 3<br>DAY 5<br>I.D.	65.7 (32.8)<br>99.0 (10.9)<br>79.9 (5.2)<br>16.3	97.3 (5.7)<br>97.9 (3.5)<br>96.1 (2.8)<br>3.0	100.5 (4.6)<br>101.4 (1.2)<br>107.7 (6.3)<br>2.5	103.7 (8.7)<br>103.2 (2.8)<br>116.6 (5.9)<br>5.8	98.0 (0.9)<br>100.3 (1.6)<br>109.5 (9.1)<br>3.9	100.8 (0.9)<br>99.8 (0.5)<br>99.5 (3.4)<br>1.6
**xanthurenic acid**	107.1	108.7	90.7	122.1	87.3	80.0	DAY 1<br>DAY 3<br>DAY 5<br>I.D.	101.5 (5.9)<br>116.5 (2.1)<br>102.2 (2.1)<br>3.3	118.8 (1.5)<br>107.4 (1.1)<br>112.7 (1.9)<br>1.5	119.7 (1.6)<br>117.4 (0.5)<br>115.2 (4.5)<br>2.2	109.9 (1.9)<br>90.6 (1.8)<br>84.8 (8.4)<br>4.0	110.6 (0.5)<br>97.6 (5.9)<br>98.0 (0.0)<br>2.1	87.9 (1.1)<br>101.1 (1.1)<br>99.6 (1.8)<br>1.3
**kynurenic acid-d_5**	98.1	108.9	80.0	108.3	103.4	91.6	DAY 1<br>DAY 3<br>DAY 5<br>I.D.	-<br>-<br>-<br>-	102.0 (1.6)<br>80.9 (2.8)<br>78.6 (4.4)<br>2.9	102.6 (9.7)<br>127.5 (1.2)<br>122.6 (1.9)<br>4.3	-<br>-<br>-<br>-	102.9 (2.6)<br>101.0 (2.2)<br>103.4 (0.3)<br>1.7	83.5 (1.2)<br>98.1 (0.7)<br>100.1 (1.6)<br>1.2
**kynurenic acid**	105.4	120.6	84.7	88.4	102.0	104.3	DAY 1	108.0 (4.1)	108.9 (3.9)	100.5 (9.6)	90.9 (1.6)	104.6 (1.7)	82.1 (1.3)

							DAY 3<br>DAY 5<br>I.D.	102.7 (1.3)<br>98.2 (1.7)<br>2.4	100.5 (1.9)<br>103.1 (7.9)<br>4.6	101.3 (0.3)<br>117.4 (2.5)<br>4.1	85.4 (2.6)<br>63.9 (0.5)<br>1.6	104.1 (3.9)<br>99.3 (1.9)<br>2.5	96.1 (0.9)<br>101.5 (2.2)<br>1.5
**tryptamine**	83.6	88.6	79.5	53.7	86.6	90.5	DAY 1<br>DAY 3<br>DAY 5<br>I.D.	66.5 (17.4)<br>88.1 (6.1)<br>54.3 (6.1)<br>10.0	93.0 (5.3)<br>101.7 (1.8)<br>91.8 (3.9)<br>3.7	91.6 (4.5)<br>101.0 (0.3)<br>91.2 (2.1)<br>2.3	119.7 (1.9)<br>107.4 (2.3)<br>94.8 (15.0)<br>6.4	108.8 (3.0)<br>102.1 (1.5)<br>108.9 (2.3)<br>2.2	96.4 (1.1)<br>93.1 (1.0)<br>101.6 (2.8)<br>1.6
**5-methoxytryptamine**	86.6	97.4	89.5	118.4	105.6	104.4	DAY 1<br>DAY 3<br>DAY 5<br>I.D.	34.0 (24.0)<br>88.8 (13.1)<br>40.3 (25.8)<br>21.0	82.6 (5.9)<br>93.5 (1.7)<br>91.8 (3.9)<br>3.8	98.2 (1.2)<br>99.4 (0.3)<br>91.0 (0.8)<br>0.7	84.8 (20.2)<br>110.6 (11.5)<br>87.5 (12.0)<br>14.6	108.3 (1.4)<br>106.4 (4.7)<br>105.1 (0.0)<br>2.1	82.8 (1.3)<br>90.4 (1.4)<br>99.7 (1.6)<br>1.4
**5-hydroxyindole acetic acid-d$_5$**	113.4	119.0	109.4	106.7	95.4	86.6	DAY 1<br>DAY 3<br>DAY 5<br>I.D.	-<br>-<br>-<br>-	103.3 (5.0)<br>98.3 (6.7)<br>98.4 (3.9)<br>5.2	99.4 (2.2)<br>99.2 (3.2)<br>103.4 (4.0)<br>3.1	-<br>-<br>-<br>-	104.4 (2.6)<br>96.5 (1.2)<br>106.9 (5.6)<br>3.1	82.5 (3.4)<br>97.1 (1.7)<br>97.9 (1.0)<br>2.0
**5-hydroxyindole acetic acid**	93.3	97.8	80.8	84.6	113.8	108.4	DAY 1<br>DAY 3<br>DAY 5<br>I.D.	107.3 (20.3)<br>91.1 (3.9)<br>92.8 (11.5)<br>11.9	107.3 (4.4)<br>102.1 (4.1)<br>92.1 (4.8)<br>4.4	104.8 (16.6)<br>127.5 (0.8)<br>104.2 (4.4)<br>7.2	74.7 (0.2)<br>109.3 (0.6)<br>97.3 (3.5)<br>1.4	102.1 (1.4)<br>91.5 (1.8)<br>91.3 (1.6)<br>1.6	97.3 (0.4)<br>113.1 (0.1)<br>90.5 (1.6)<br>0.7
**N-acetyl-5-hydroxytryptamine**	84.4	105.9	100.3	132.5	118.7	113.8	DAY 1<br>DAY 3<br>DAY 5<br>I.D.	104.9 (7.5)<br>108.3 (13.3)<br>95.5 (7.6)<br>9.5	102.1 (3.4)<br>103.4 (4.9)<br>93.0 (5.4)<br>4.6	126.6 (1.8)<br>110.6 (1.3)<br>99.9 (3.3)<br>2.1	101.2 (6.5)<br>92.6 (6.8)<br>104.4 (8.5)<br>7.3	95.3 (3.4)<br>103.5 (4.0)<br>103.8 (1.4)<br>2.9	73.2 (2.8)<br>87.3 (1.4)<br>99.4 (2.0)<br>3.0
**tryptophan methyl ester**	88.1	95.0	86.7	83.4	83.4	81.4	DAY 1<br>DAY 3<br>DAY 5<br>I.D.	91.0 (26.9)<br>89.0 (5.7)<br>78.1 (24.1)<br>18.9	88.8 (3.3)<br>86.4 (1.4)<br>93.0 (1.9)<br>2.2	96.7 (3.7)<br>92.9 (2.3)<br>101.4 (1.8)<br>2.6	79.5 (20.7)<br>110.5 (7.1)<br>88.6 (9.9)<br>12.6	110.7 (2.2)<br>125.9 (8.1)<br>108.9 (1.1)<br>3.8	80.0 (2.3)<br>86.9 (1.9)<br>95.7 (0.5)<br>1.6
**homovanillic acid**	-	93.7	92.9	104.5	106.2	94.0	DAY 1<br>DAY 3<br>DAY 5<br>I.D.	-<br>-<br>-<br>-	95.2 (11.9)<br>96.7 (14.3)<br>95.5 (5.6)<br>14.6	100.2 (1.7)<br>100.9 (2.2)<br>104.1 (4.8)<br>2.9	163.8 (1.5)<br>171.5 (3.7)<br>35.7 (3.4)<br>2.9	104.2 (2.6)<br>116.4 (3.5)<br>99.0 (3.0)<br>3.0	73.9 (16.5)<br>90.7 (6.8)<br>98.7 (1.2)<br>8.1
**indoxyl sulfate**	102.9	99.4	96.9	101.8	98.4	91.5	DAY 1<br>DAY 3<br>DAY 5<br>I.D.	121.2 (1.2)<br>81.9 (1.2)<br>83.7 (2.6)<br>1.7	94.2 (3.4)<br>88.5 (3.0)<br>98.0 (2.7)<br>3.0	105.5 (2.5)<br>104.4 (2.3)<br>106.6 (6.4)<br>3.7	93.4 (0.3)<br>84.0 (0.6)<br>126.3 (0.1)<br>0.3	124.9 (0.7)<br>87.2 (0.2)<br>105.2 (0.4)<br>0.5	95.4 (3.8)<br>77.7 (4.9)<br>91.4 (2.1)<br>3.6
**indole-3-acetamide**	90.1	103.0	89.8	87.5	114.2	105.4	DAY 1<br>DAY 3<br>DAY 5<br>I.D.	94.4 (6.0)<br>102.0 (10.2)<br>102.8 (17.2)<br>11.1	102.2 (3.5)<br>104.2 (3.6)<br>97.5 (2.6)<br>3.2	107.8 (4.8)<br>105.4 (4.3)<br>97.9 (4.1)<br>4.4	92.0 (6.1)<br>105.1 (5.2)<br>83.2 (9.6)<br>7.0	98.6 (5.0)<br>101.9 (9.1)<br>109.8 (0.3)<br>4.8	86.7 (5.8)<br>93.5 (1.8)<br>100.6 (1.1)<br>1.8
**anthranilic acid**	91.5	89.0	90.1	93.4	87.9	97.1	DAY 1<br>DAY 3<br>DAY 5<br>I.D.	67.5 (12.9)<br>106.2 (6.1)<br>74.3 (13.0)<br>10.6	80.0 (4.3)<br>92.3 (3.0)<br>78.8 (5.9)<br>4.4	99.4 (4.4)<br>101.0 (3.1)<br>89.5 (3.7)<br>3.6	101.5 (2.6)<br>100.5 (6.3)<br>82.1 (2.9)<br>4.0	113.4 (2.5)<br>100.4 (7.2)<br>110.5 (1.1)<br>3.6	101.1 (1.1)<br>91.7 (0.9)<br>98.3 (2.1)<br>1.4
**indole-3-lactic acid**	99.9	102.8	91.1	72.5	98.9	97.2	DAY 1<br>DAY 3<br>DAY 5<br>I.D.	104.0 (3.5)<br>104.2 (1.1)<br>109.7 (6.0)<br>3.5	102.4 (2.7)<br>100.2 (0.2)<br>96.2 (5.1)<br>2.7	110.7 (2.0)<br>100.0 (0.6)<br>102.0 (4.1)<br>2.3	102.1 (1.2)<br>110.6 (2.9)<br>100.7 (0.6)<br>1.6	103.3 (1.8)<br>99.7 (5.5)<br>113.4 (6.3)<br>4.5	104.5 (2.3)<br>97.0 (1.7)<br>100.6 (0.6)<br>1.5
**indole-3-carboxylic acid**	77.9	85.4	113.2	93.2	85.2	96.8	DAY 1<br>DAY 3<br>DAY 5<br>I.D.	103.4 (16.5)<br>101.5 (17.7)<br>103.0 (7.5)<br>13.9	107.8 (6.7)<br>108.3 (2.6)<br>95.9 (5.8)<br>5.0	105.2 (5.3)<br>104.9 (2.0)<br>95.5 (4.7)<br>4.0	116.0 (18.3)<br>124.9 (2.4)<br>77.4 (20.5)<br>13.7	85.3 (6.5)<br>95.7 (1.7)<br>100.4 (2.9)<br>3.7	91.8 0.2<br>86.6 (1.1)<br>89.6 (2.7)<br>1.3
**melatonin**	88.7	97.9	85.2	114.	111.0	103.9	DAY 1<br>DAY 3<br>DAY 5<br>I.D.	84.6 (11.7)<br>98.4 (4.3)<br>105.3 (1.4)<br>5.8	99.2 (1.8)<br>99.0 (0.7)<br>96.1 (2.6)<br>1.7	102.7 (4.6)<br>110.5 (2.8)<br>102.7 (1.8)<br>3.1	116.4 (3.9)<br>127.0 (3.8)<br>114.0 (2.0)<br>3.2	99.8 (1.6)<br>107.8 (10.9)<br>103.7 (2.0)<br>7.7	94.1 (1.9)<br>83.3 (1.3)<br>92.3 (0.5)<br>1.2
**5-methoxyindole acetic acid**	93.7	104.0	92.0	133.2	109.4	109.1	DAY 1<br>DAY 3<br>DAY 5<br>I.D.	102.9 (16.5)<br>81.1 (8.1)<br>101.9 (16.8)<br>13.8	104.4 (2.7)<br>101.9 (0.5)<br>99.4 (5.3)<br>2.8	104.6 (1.8)<br>109.7 (0.8)<br>102.7 (3.2)<br>1.9	75.8 (10.6)<br>90.9 (10.8)<br>74.1 (13.6)<br>11.7	97.6 (1.8)<br>84.8 (2.5)<br>105.8 (3.1)<br>2.4	89.2 (3.6)<br>91.4 (1.8)<br>96.9 (0.7)<br>1.0
**indole-3-carboxaldehyde**	109.5	93.7	84.5	117.1	94.9	87.6	DAY 1<br>DAY 3<br>DAY 5<br>I.D.	119.8 (3.1)<br>98.3 (4.1)<br>94.3 (3.5)<br>3.6	112.5 (1.4)<br>103.2 (2.1)<br>102.4 (2.6)<br>2.0	103.5 (4.5)<br>103.3 (0.4)<br>101.5 (3.9)<br>2.9	91.8 (2.6)<br>98.8 (4.1)<br>94.2 (2.3)<br>3.0	100.9 (0.7)<br>101.0 (5.3)<br>101.3 (2.3)<br>2.8	93.4 (0.5)<br>89.5 (0.9)<br>97.1 (0.4)<br>0.6

**indole-3-acetonitrile**	101.7	85.5	95.6	112.7	113.7	105.9	DAY 1<br>DAY 3<br>DAY 5<br>I.D.	122.6 (3.6)<br>109.5 (0.1)<br>128.9 (3.9)<br>2.5	110.9 (0.9)<br>103.5 (2.2)<br>105.4 (4.8)<br>2.6	101.4 (5.6)<br>102.1 (2.0)<br>105.1 (2.6)<br>3.4	93.5 (1.7)<br>77.4 (0.8)<br>79.9 (1.6)<br>1.4	102.3 (0.3)<br>110.7 (2.5)<br>100.2 (3.6)<br>2.1	95.5 (1.0)<br>76.5 (1.7)<br>91.0 (2.2)<br>1.6
**indole-3-acetic acid**	101.7	86.5	97.9	86.1	107.3	106.7	DAY 1<br>DAY 3<br>DAY 5<br>I.D.	113.8 (2.9)<br>114.8 (1.3)<br>112.8 (2.8)<br>2.3	111.0 (0.7)<br>108.6 (2.0)<br>103.8 (4.0)<br>2.3	101.1 (3.2)<br>109.0 (0.3)<br>104.9 (3.9)<br>2.5	113.0 (1.1)<br>77.3 (0.2)<br>109.3 (4.4)<br>1.9	103.0 (1.3)<br>102.5 (2.0)<br>103.9 (1.4)<br>1.6	97.3 (1.3)<br>88.3 (0.9)<br>101.7 (1.7)<br>1.3
**indole-3-propionic acid**	89.0	102.2	89.3	94.0	101.3	106.0	DAY 1<br>DAY 3<br>DAY 5<br>I.D.	101.8 (2.5)<br>104.1 (1.7)<br>104.7 (4.2)<br>2.8	103.5 (1.5)<br>104.3 (1.9)<br>94.4 (1.6)<br>1.7	105.9 (3.5)<br>103.5 (0.4)<br>101.1 (2.1)<br>2.0	95.0 (1.9)<br>103.7 (4.9)<br>100.9 (1.9)<br>2.9	97.9 (1.2)<br>98.9 (6.2)<br>104.3 (7.0)<br>4.8	98.6 (0.7)<br>101.6 (0.9)<br>102.1 (1.3)<br>1.1

Table S3. Stock solution (ng/ml) and concentrations (low, medium and high) used for recovery and accuracy assays in plasma and urine.

	PLASMA				URINE			
**Metabolite**	**Stock solution (ng/ml)**	**LOW (ng/ml)**	**MED (ng/ml)**	**HIGH (ng/ml)**	**Stock solution (ng/ml)**	**LOW (ng/ml)**	**MED (ng/ml)**	**HIGH (ng/ml)**
**γ-aminobutyric acid**	25000	50	500	5000	25000	25	250	2500
**L-valine**	50000	100	1000	10000	50000	50	500	5000
**picolinic acid**	25000	50	500	5000	25000	25	250	2500
**dopamine-d$_4$**	12500	25	250	2500	12500	12.5	125	1250
**dopamine**	2000	4	40	400	2000	2	20	200
**methionine-d$_4$**	12500	25	250	2500	12500	12.5	125	1250
**methionine**	25000	50	500	5000	25000	25	250	2500
**2-aminophenol**	2000	4	40	400	2000	2	20	200
**quinolinic acid**	25000	50	500	5000	25000	25	250	2500
**3-hydroxykynurenine**	2000	4	40	400	2000	2	20	200
**tyrosine-d$_4$**	12500	25	250	2500	12500	12.5	125	1250
**tyrosine**	25000	100	1000	10000	50000	50	500	5000
**L-isoleucine**	50000	100	1000	10000	50000	50	500	5000
**tyramine**	2000	4	40	400	2000	2	20	200
**L-leucine**	50000	100	1000	10000	50000	50	500	5000
**serotonin-d$_4$**	12500	25	250	2500	12500	12.5	125	1250
**serotonin**	2000	4	40	400	2000	2	20	200
**5-hydroxy-tryptophan**	12500	4	40	400	12500	12.5	125	1250
**3-methoxy-*p*-tyramine**	2000	4	40	400	2000	2	20	200
**kynurenine**	25000	50	500	5000	25000	25	250	2500
**DL-phenylalanine**	25000	50	500	5000	50000	50	500	5000
**3-hydroxyanthranilic acid**	2000	4	40	400	2000	2	20	200
**tryptophan-d$_5$**	12500	25	250	2500	12500	12.5	125	1250
**tryptophan**	50000	100	1000	10000	50000	50	500	5000
**1-acetylisatin**	2000	4	40	400	2000	2	20	200
**3,4-dihydroxyphenyl acetic acid-d$_5$**	25000	50	500	5000	25000	25	250	2500
**3,4-dihydroxyphenyl acetic acid**	25000	50	500	5000	25000	25	250	2500
**xanthurenic acid**	25000	50	500	5000	25000	25	250	2500
**kynurenic acid-d$_5$**	12500	25	250	2500	12500	12.5	125	1250
**kynurenic acid**	25000	50	500	5000	25000	25	250	2500
**tryptamine**	2000	4	40	400	2000	2	20	200
**5-methoxytryptamine**	2000	4	40	400	2000	2	20	200
**5-hydroxyindole acetic acid-d$_5$**	12500	25	250	2500	12500	12.5	125	1250
**5-hydroxyindole acetic acid**	2000	4	40	400	2000	2	20	200
**N-acetyl-5-hydroxytryptamine**	2000	4	40	400	2000	2	20	200
**tryptophan methyl ester**	2000	4	40	400	2000	2	20	200
**homovanillic acid**	25000	50	500	5000	25000	25	250	2500
**indoxyl sulfate**	25000	50	500	5000	25000	25	250	2500
**indole-3-acetamide**	2000	4	40	400	2000	2	20	200
**anthranilic acid**	2000	4	40	400	2000	2	20	200
**indole-3-lactic acid**	25000	50	500	5000	25000	25	250	2500
**indole-3-carboxylic acid**	2000	4	40	400	2000	2	20	200

**melatonin**	2000	4	40	400	2000	2	20	200
**5-methoxyindole acetic acid**	2000	4	40	400	2000	2	20	200
**indole-3-carboxaldehyde**	2000	4	40	400	2000	2	20	200
**indole-3-acetonitrile**	25000	50	500	5000	25000	25	250	2500
**indole-3-acetic acid**	25000	50	500	5000	25000	25	250	2500
**indole-3-propionic acid**	25000	50	500	5000	25000	25	250	2500

Table S4. Metabolite concentration ranges detected in plasma and urine compared to reference data. n.d.: not detected; n.a.: not available.

	Concentration			
**Metabolite**	**Plasma (ng/ml)**	**Reference**	**Urine (ng/ml)**	**Reference**
**L-valine**	1426-15394	11839-37830 ng/mM creatinine [50]	326-18449	1262-12424 ng/mg creatinine [48]<br>234-902 ng/mM creatinine [51]
**picolinic acid**	0.2-7.1	32-40 ng/ml[52]	132-509	886-4628 ng/mM creatinine [51]
**dopamine**	2-11	10 ng/ml [53]	46-1278	48-812 ng/mg creatinine [48]
**methionine**	451-3769	3506-5386 ng/mM creatinine [50]	0.25-8754	1318-6592 ng/mg creatinine [48]<br>74-373 ng/mM creatinine [51]
**2-aminophenol**	n.d.	-	n.d.	-
**quinolinic acid**	69-1630	-	2938-40456	167-2924 ng/mM creatinine [51]
**3-hydroxykynurenine**	-	-	38-1359	224-762 umol/mmol creatinine [53]
**tyrosine**	1224-15056	8117-11632 ng/mM creatinine [50]	702-15352	2715-57663 ng/mg creatinine [48]<br>742-4257 ng/mM creatinine [51]
**L-isoleucine**	650-10297	5522-10401 ng/mM creatinine [50]	22-11909	1158-10433 ng/mg creatinine[48]<br>52-341 ng/mM creatinine [51]
**tyramine**	n.d.	1-1.6 ng/ml [53]	27-109	49-96 ng/ml [54]
**L-leucine**	1414-15804	11438-14454 ng/mM creatinine[50]	384-34525	2318-18548 ng/mg creatinine[48]<br>209-708 ng/mM creatinine [51]
**serotonin**	0.1-185	81-179 ng/ml [53]	10-482	15-366 ng/mg creatinine [48]
**5-hydroxy-tryptophan**	n.d.	3-5 ng/ml [55]	16-179	0.0078 umol/mmol creatinine [54]
**3-methoxy-*p*-tyramine**	n.d.	-	4-287	19-80 ng/mg creatinine [48]
**kynurenine**	94-727	395-603 ng/ml [53]	66-13511	202-1988 ng/mg creatinine [48]<br>229-520 ng/mM creatinine [51]
**DL-phenylalanine**	1345-11851	9514-16287 ng/mM creatinine [50]	1717-87803	2145-18982 ng/mg creatinine [48] 578-1850 ng/mM creatinine [51]
**3-hydroxyanthranilic acid**	27-49	2-21 ng/ml[56]	27-1852	10-2957 ng/mg creatinine [48]
**tryptophan**	1744-16729	9149-13111 ng/mM creatinine [50]	2129-110628	3060-25274 ng/mg creatinine [48]694-2266 ng/mM creatinine [51]
**1-acetylisatin**	n.d.	-	n.d.	-
**3,4-dihydroxyphenyl acetic acid**	2-12317	1.6-3.3 ng/ml [57]	n.d.	1.16 +-0.50 umol/mmol creatinine[53]
**xanthurenic acid**	4-37.6	4.3-4.7 ng/ml [58]	118-10955	481-1634 ng/mg creatinine[48]
**kynurenic acid**	1-31	2.45-6.24 ng/ml [56]	810-26581	551-6053 ng/mg creatinine [48]<br>151-794 ng/mM creatinine [51]
**tryptamine**	n.d.	-	10-979	-
**5-methoxytryptamine**	n.d.	-	n.d.	0-0.6 ng/ml (HMDB)
**5-hydroxyindole acetic acid**	3-87	8.5-11 ng/ml [59]	6-2329	169-5696 ng/mg creatinine[48]<br>91-860 umol/mmol creatinine [53]
**N-acetyl-5-hydroxytryptamine**	n.d.	1.1 ng/ml [60]	n.d.	-
**tryptophan methyl ester**	n.d.	-	n.d.	-
**homovanillic acid**	2-181	5-24 ng/ml[59]	1216-24765	2461-16106 ng/mg creatinine[48]<br>327-2313 ng/mM creatinine [51]
**indoxyl sulfate**	10-2754	2089-3880 ng/ml [53]	n.a.	1279-13816 ng/mM creatinine [51]
**indole-3-acetamide**	n.d.	-	4-2595	-
**anthranilic acid**	n.d.	-	16-502	2.6-5.85 ng/ml [61]
**indole-3-lactic acid**	15-826	87-875 ng/ml [53]	60-5731	0.098-0.98 umol/mmol creatinine [53]
**indole-3-carboxylic acid**	n.d.	-	10-1993	-
**melatonin**	n.d.	0.13-0.14 ng/ml [53]	n.d.	0.0000033 umol/mmol creatinine [53]
**5-methoxyindole acetic acid**	n.d.	-	n.d.	-
**indole-3-carboxaldehyde**	1.5-27	-	0.7-864	-
**indole-3-acetonitrile**	42-4130	-	566-22366	-
**indole-3-acetic acid**	51-4051	199-798 ng/ml [61]	1319-53106	315-1086 ng/mM creatinine[51]
**indole-3-propionic acid**	2-2424	55-207 ng/ml [62]	5-614	-

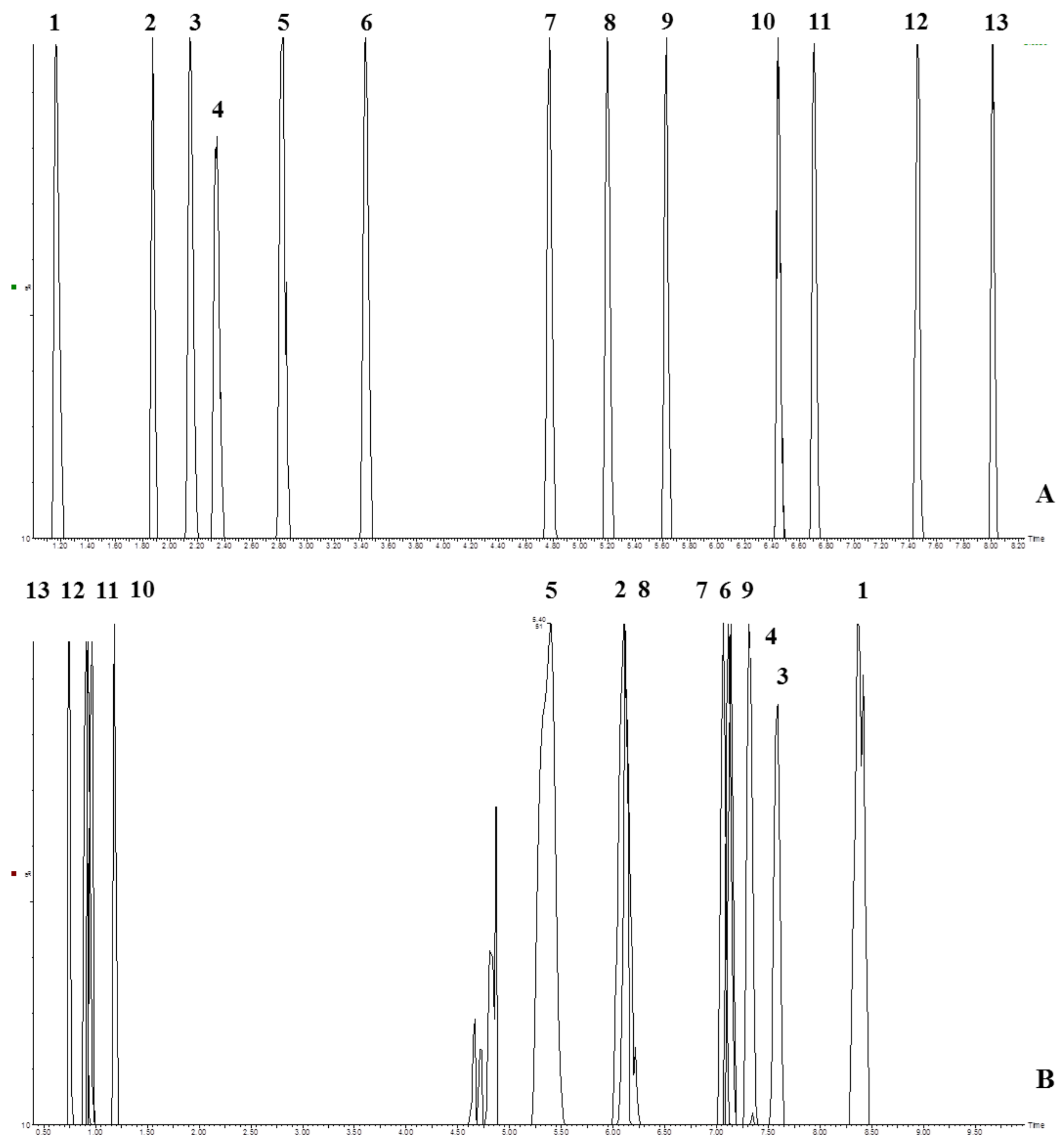

Figure S1. Chromatograms of 13 standard compounds obtained by RP chromatography on Waters HSST3 1.8 µm, 2.1 x 150 mm column (panel A) and by HILIC on a Waters BEH AMIDE 1.7 µm, 2.1 x 150 mm (B). Legend: 1: GABA; 2: DA; 3: ILE; 4: LEU; 5: 5-HT; 6: PHE; 7: TRP; 8: KA; 9: 5OH-IAA; 10: IACT; 11: AA; 12: IACN; 13: IPA

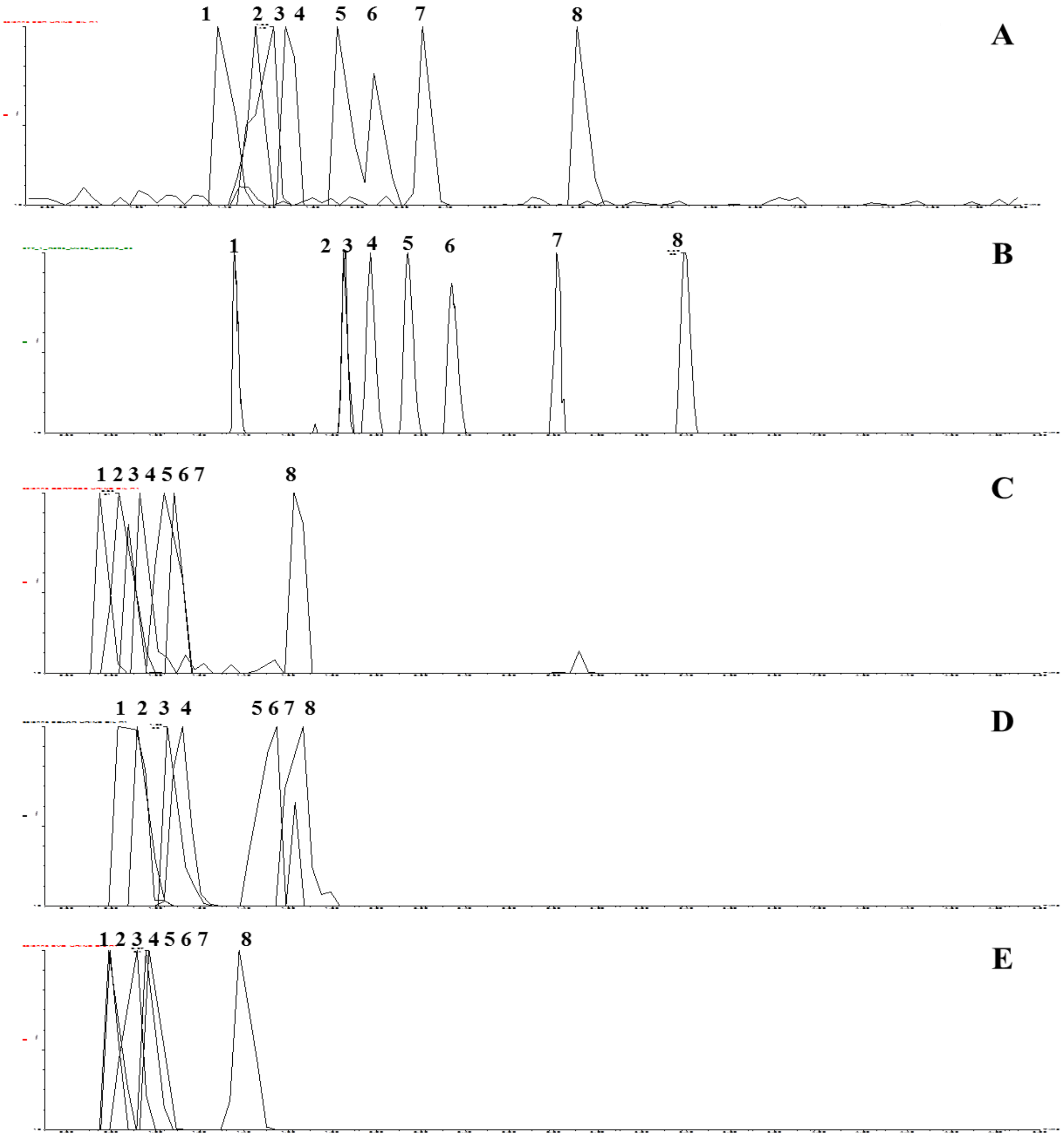

Figure S2. Chromatograms of the most polar metabolites obtained on A) Waters ACQUITY BEH C_{18} 1.7 µm, 2.1 x 150 mm; B) Waters ACQUITY HSST3 1.8 µm, 2.1 x 150 mm; C) Waters Cortecs UPLC C_{18} 1.6 µm, 2.1 x 100 mm; D) Phenomenex Kinetex Polar C_{18} 2.6 µm, 2.1 x 100 mm; E) Phenomenex Kinetex EVO C_{18} 2.6 µm, 2.1 x 100 mm. Peaks: 1) VAL; 2) MET; 3) DA; 4) TYR; 5) ILE; 6) LEU; 7) 5-HT; 8) PHE
